# Supplementary material for: Integrating single cell expression quantitative trait loci summary statistics to understand complex trait risk genes
Source: Nat Commun. 2024 May 20;15:4260. doi: 10.1038/s41467-024-48143-1 (PMC11519974; doi:10.1038/s41467-024-48143-1)
Supplement: Supplementary file 1 — Supplementary Information [file 41467_2024_48143_MOESM1_ESM.pdf]

Supplementary Information for

Integrating single cell expression quantitative trait loci summary statistics to understand complex trait risk genes.

Authors:

Lida Wang[1,#], Chachrit Khunsriraksakul[2,3,#], Havell Markus[2,3], Dieyi Chen[1], Fan Zhang[2], Fang Chen[1], Xiaowei Zhan[4,5,6], Laura Carrel[7,*], Dajiang J. Liu[1,2,4,*], Bibo Jiang[1,*]

1. Department of Public Health Sciences; Pennsylvania State University College of Medicine; Hershey, Pennsylvania, 17033; USA.
2. Bioinformatics and Genomics PhD Program; Pennsylvania State University College of Medicine; Hershey, Pennsylvania, 17033; USA.
3. Institute for Personalized Medicine; Pennsylvania State University College of Medicine; Hershey, Pennsylvania, 17033; USA.
4. Department of Statistical Science, Southern Methodist University, Dallas, TX 75275, United States
5. Quantitative Biomedical Research Center, Department of Population and Data Sciences, University of Texas Southwestern Medical Center, Dallas, TX 75390, United States
6. Center for Genetics of Host Defense, University of Texas Southwestern Medical Center, Dallas, TX 75390, United States
7. Department of Biochemistry and Molecular Biology; Pennsylvania State University College of Medicine; Hershey, Pennsylvania, 17033; USA.

#: these authors contributed to the work equally.

*: Manuscript correspondence should be addressed to:

Laura Carrel: lcarrel@pennstatehealth.psu.edu, or

Dajiang J. Liu: dajiang.liu@psu.edu, or

Bibo Jiang: bjiang@phs.psu.edu.

This file includes:

Supplementary Figures 1 to 9

Supplementary Methods

Supplementary References

Supplementary Figures

Supplementary Figure 1: Simulation studies comparing the performance of EXPRESSO to other TWAS methods. We compare EXPRESSO to single tissue TWAS methods using (A) median of Pearson correlation between predicted and measured gene expression; (B) proportion of genes whose predicted and measured gene expression levels are significantly correlated i.e., Pearson correlation (R)> .1 with two-sided p-value < 0.05; (C) TWAS power measured by the proportion of genes whose two-sided TWAS p-values < 0.05/the number of genes tested. We also compare EXPRESSO with multi-tissue method UTMOST.

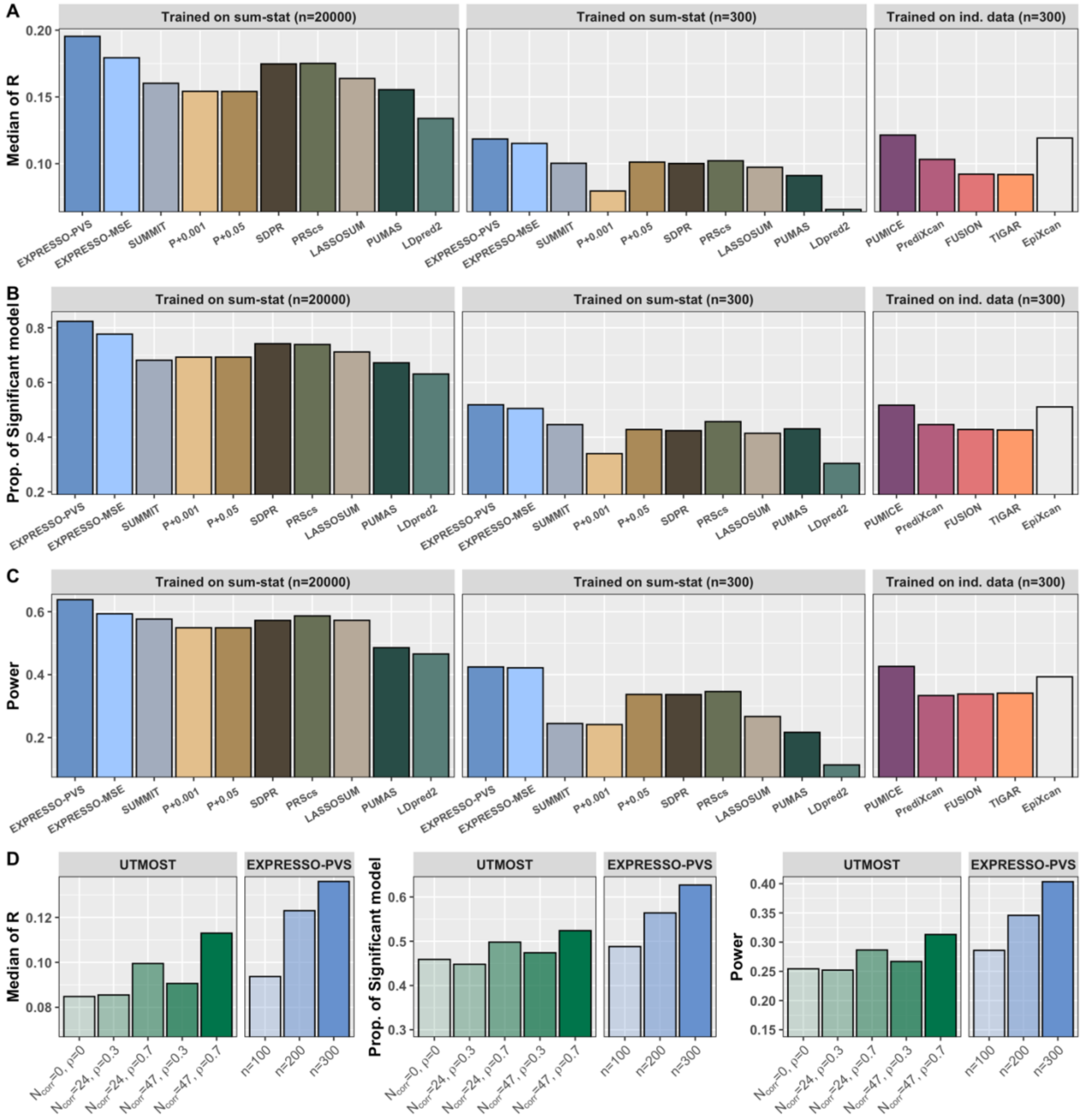

Supplementary Figure 2: Clustering the cell types. Panel A illustrates clusters of difference cell types using the principal components of the eQTL effects. Panel B displays the hierarchical cluster of eQTL effects from different cell types. Principal component analysis and hierarchical clustering yield similar and biologically informative groupings of different cell types.

A

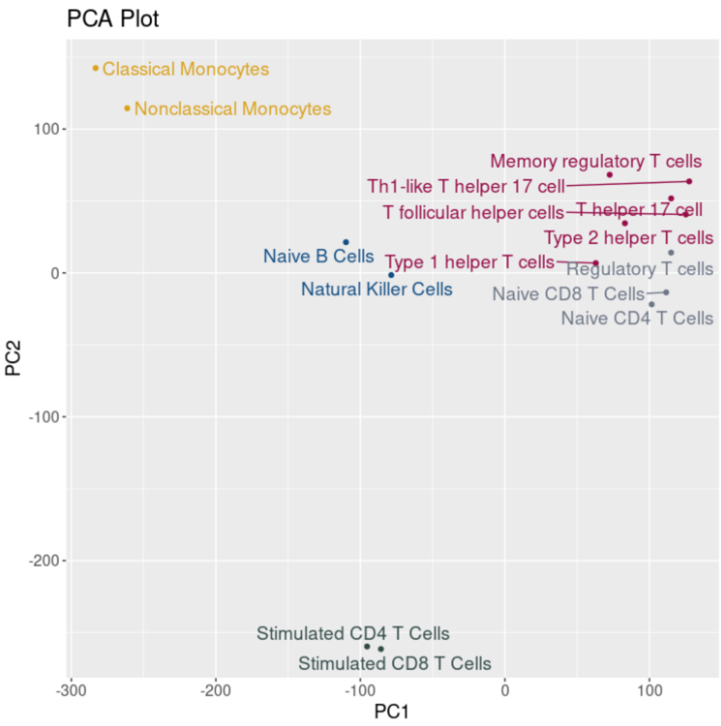

B

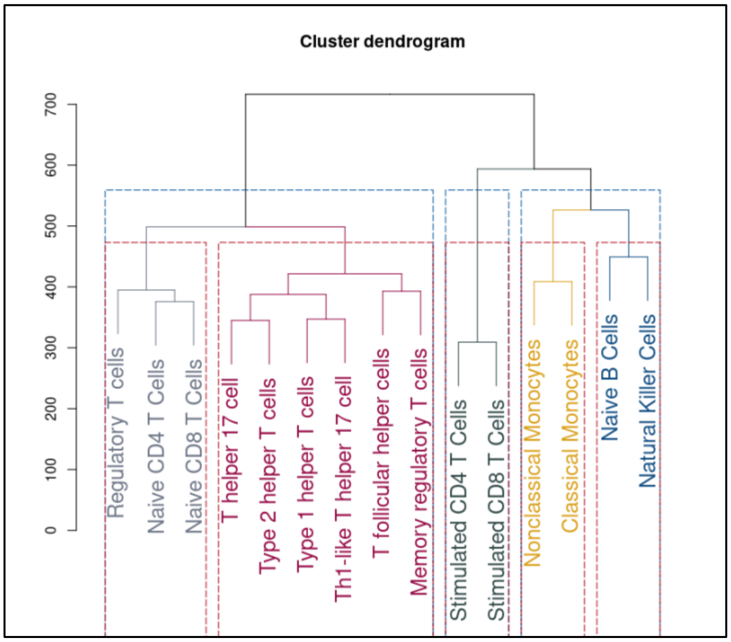

Supplementary Figure 3: Distribution of window size *w* and mitigation parameter ϕ among selected EXPRESSO-PVS models. Panel (A) depicts the distribution of various window sizes (w) among the selected EXPRESSO-PVS models. In panel (B), we present the distribution of different values of the tuning parameter ϕ among the selected EXPRESSO-PVS models. 3D genome-informed regions (loop, TAD, domain, pcHi-C) are chosen 47.27% of the time. On the other hand, the 1 million basepair window, the default window size for many methods, are only chosen 31.28% of the time. The most frequent choice for mitigation parameter is ϕ = 1/6 (31.01%), which prioritizes essential predictors by assigning much smaller L_1 and L_2 penalties.

A.

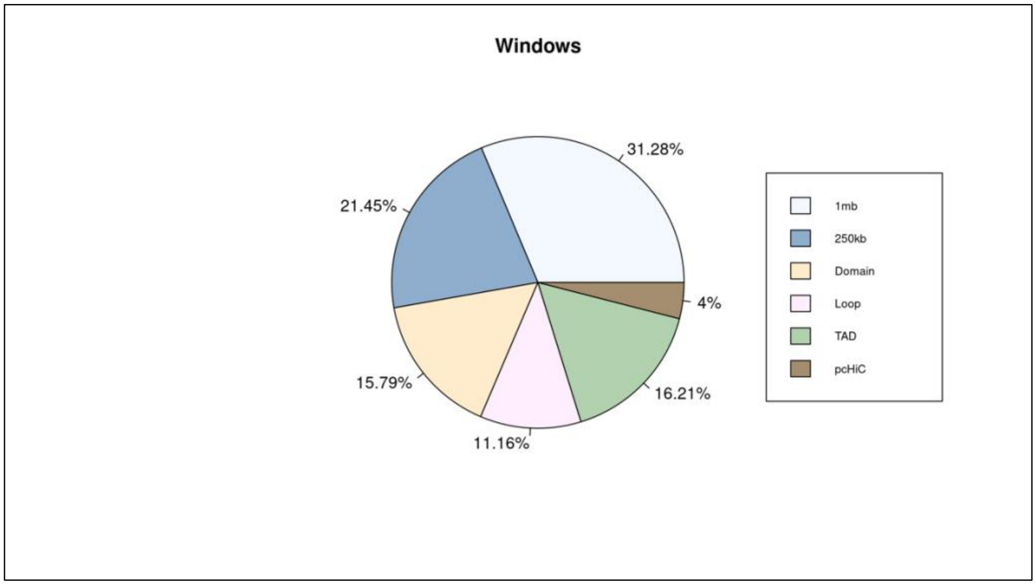

B.

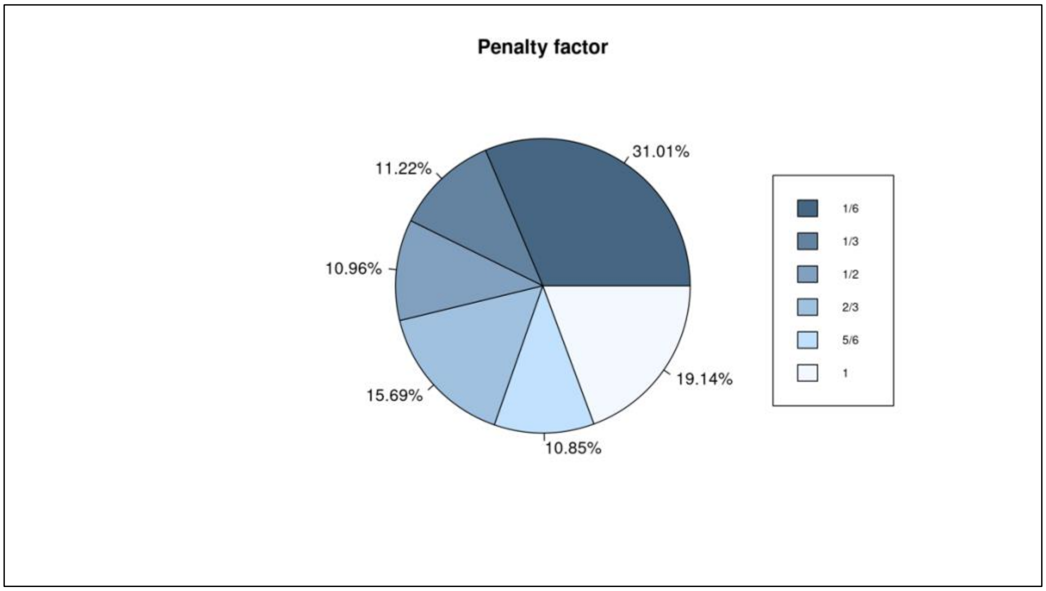

Supplementary Figure 4: Effective sample sizes across 14 autoimmune diseases. We calculate effective sample size (N_{eff}) according to the formula $2/(1/N_{cases} + 1/N_{controls})$, which is more appropriate for studies with unbalanced number of cases and controls. Colors represent sample ancestries.

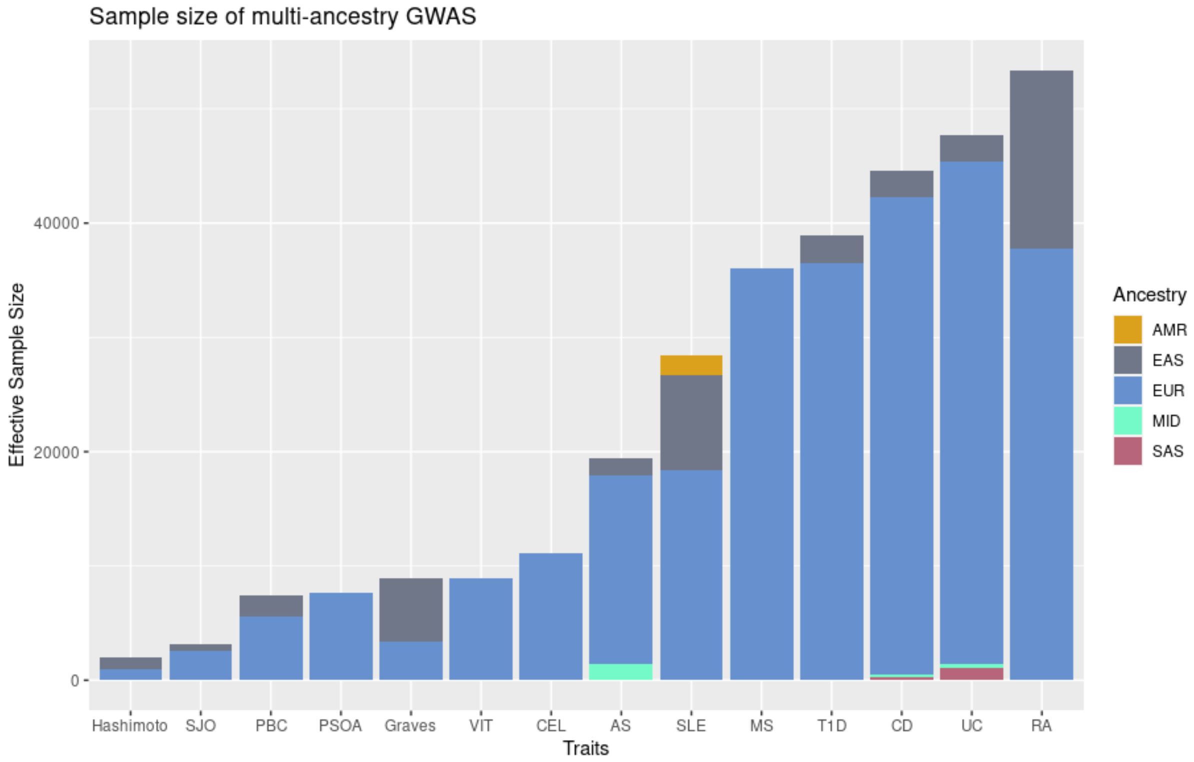

Abbreviations: AMR = Admixed American, EAS = East Asian, EUR = European, MID = Middle Eastern, SAS = South Asian

Supplementary Figure 5: Cell type enrichment analysis. Panels A-N illustrate the results for cell type enrichment analyses for 15 immune cell types from DICE dataset. For each trait, we plot the −log (two-sided p-value) for 15 cell types. We denote the nominal significance level (red dash line) and significance level with Bonferroni correction (red solid line).

A. Ankylosing spondylitis.

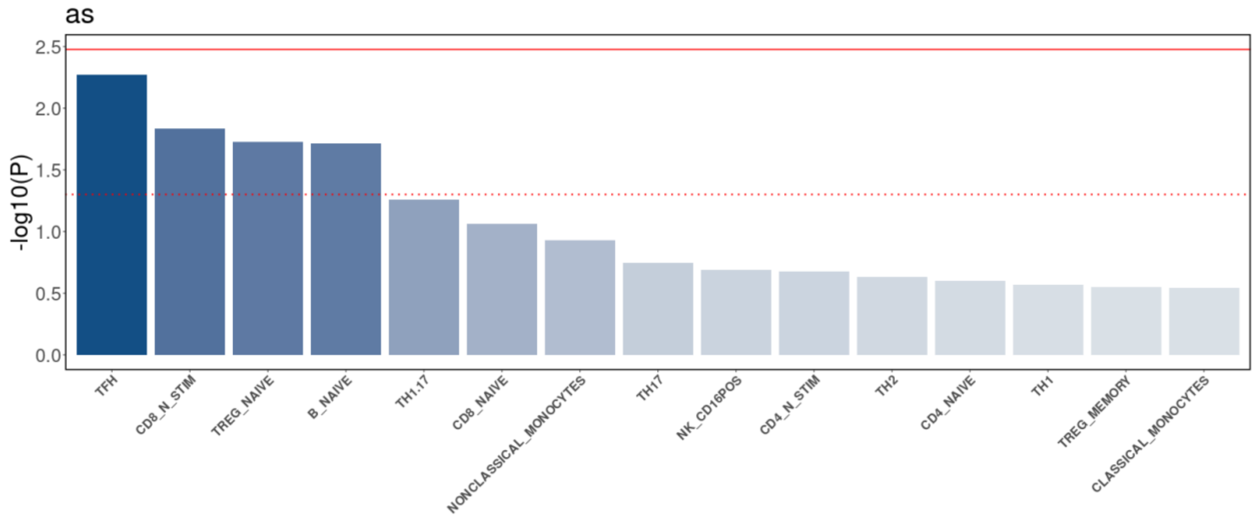

B. Crohn's disease.

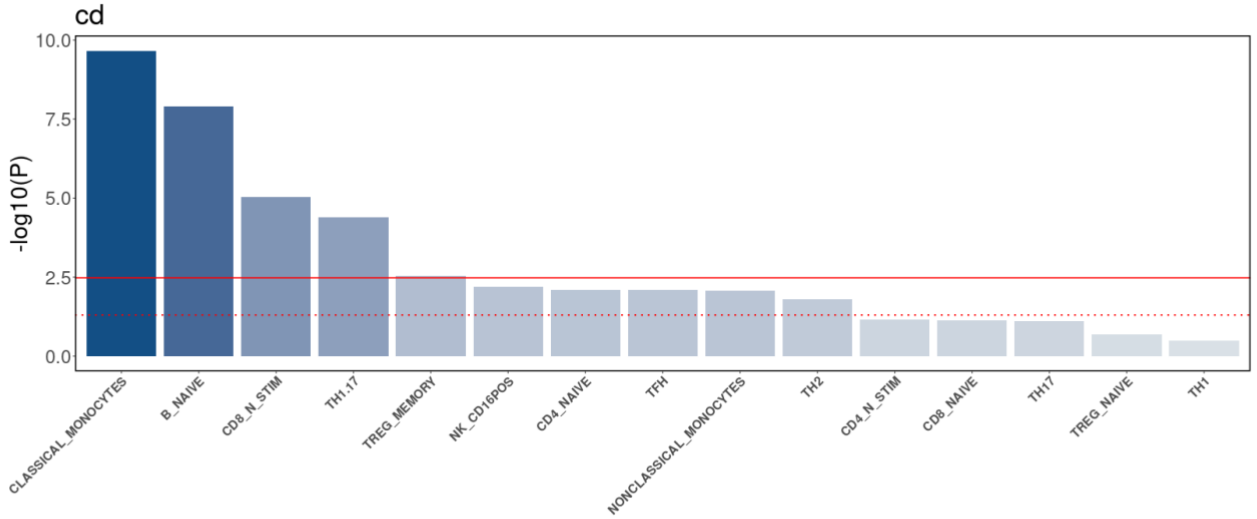

C. Celiac disease.

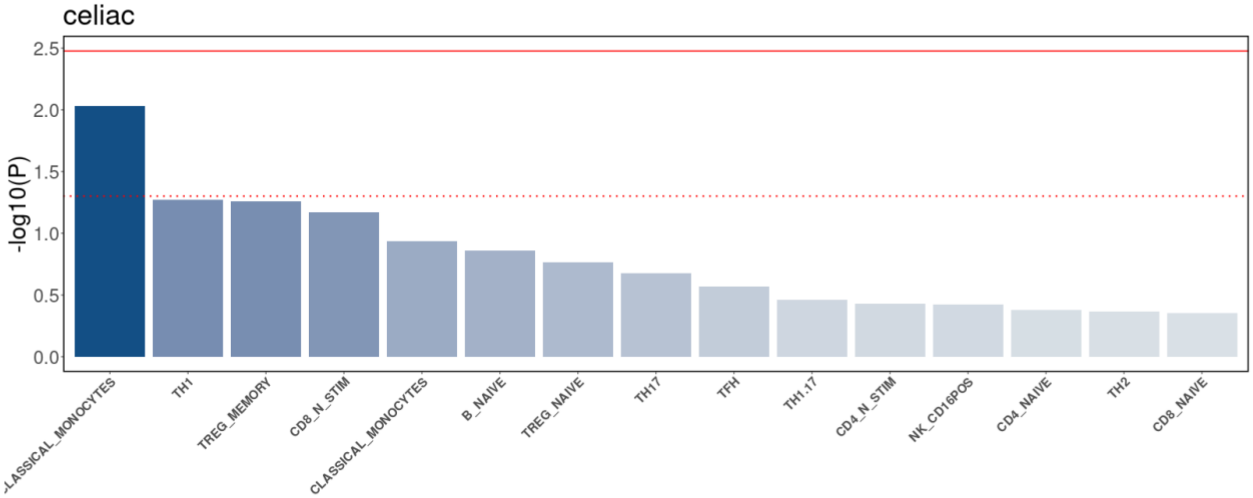

D. **ATD-Grave's disease.**

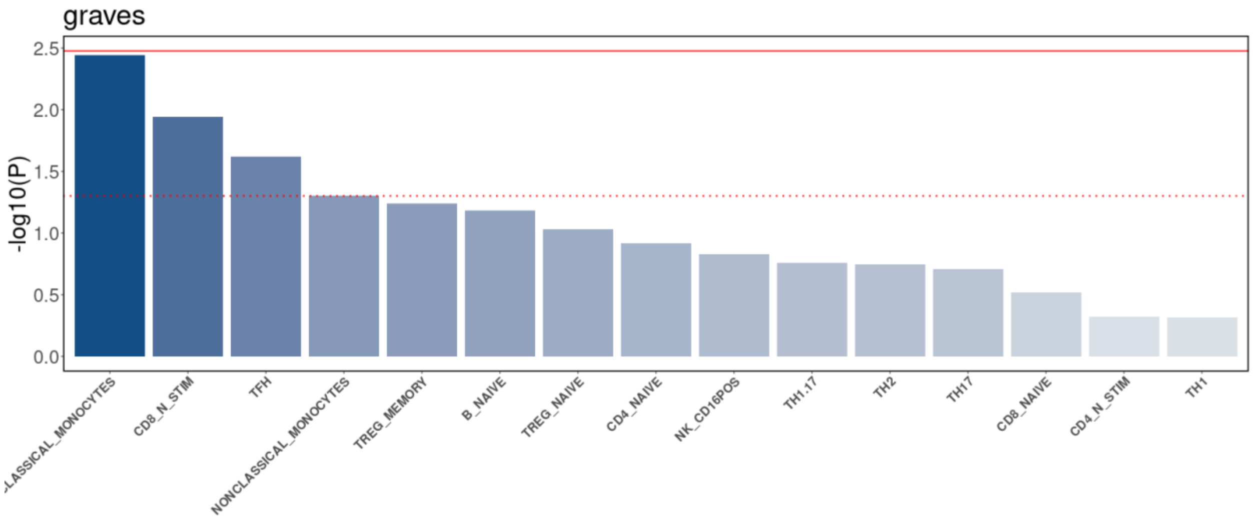

E. **ATD-Hashimoto thyroiditis.**

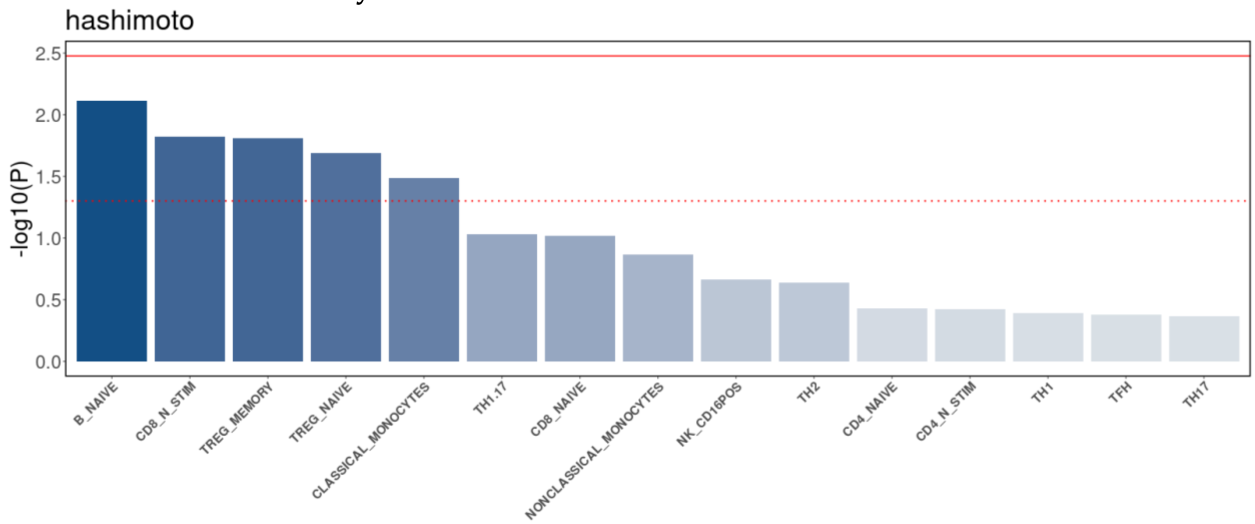

F. **Multiple sclerosis.**

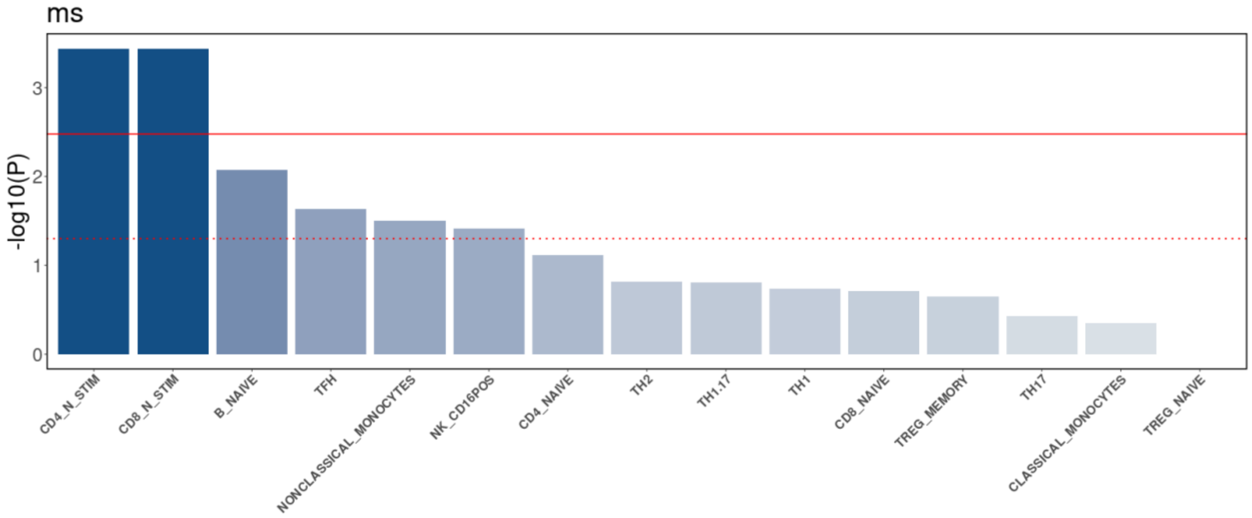

G. Primary biliary cirrhosis.

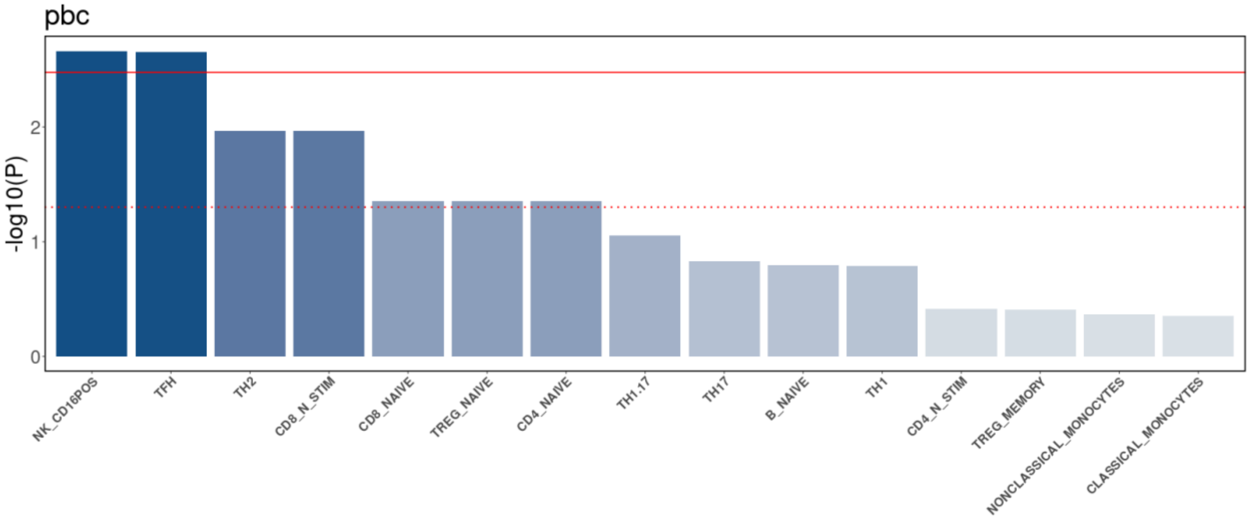

H. Psoriatic arthritis.

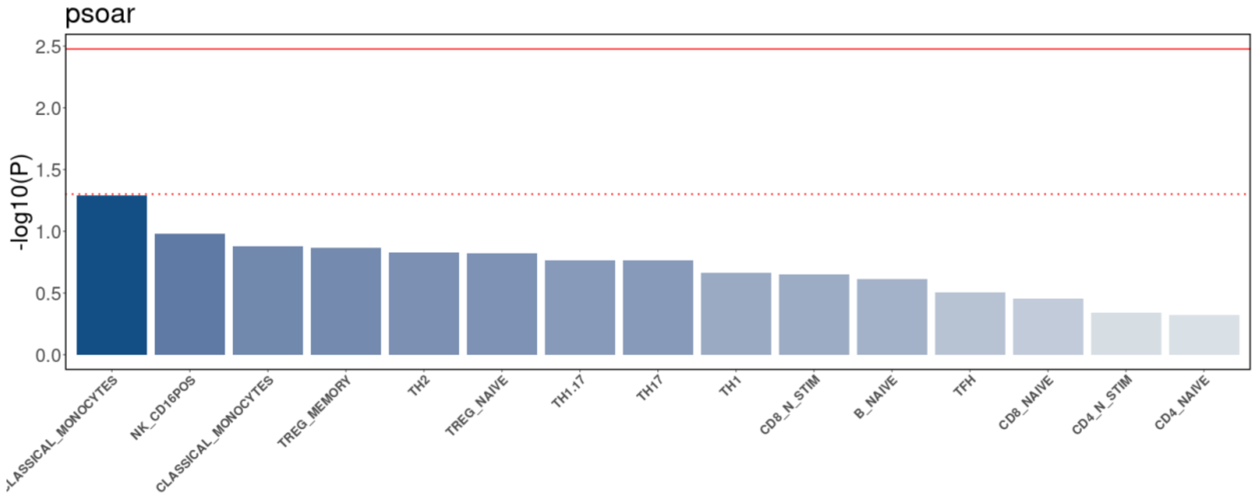

I. Rheumatoid arthritis.

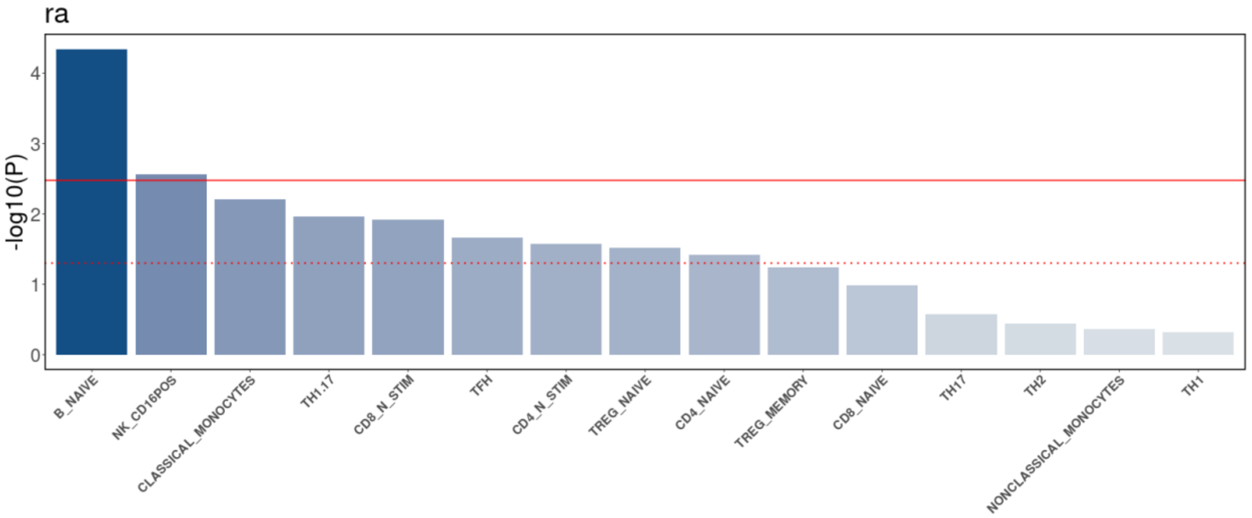

J. Sjogren's syndrome.

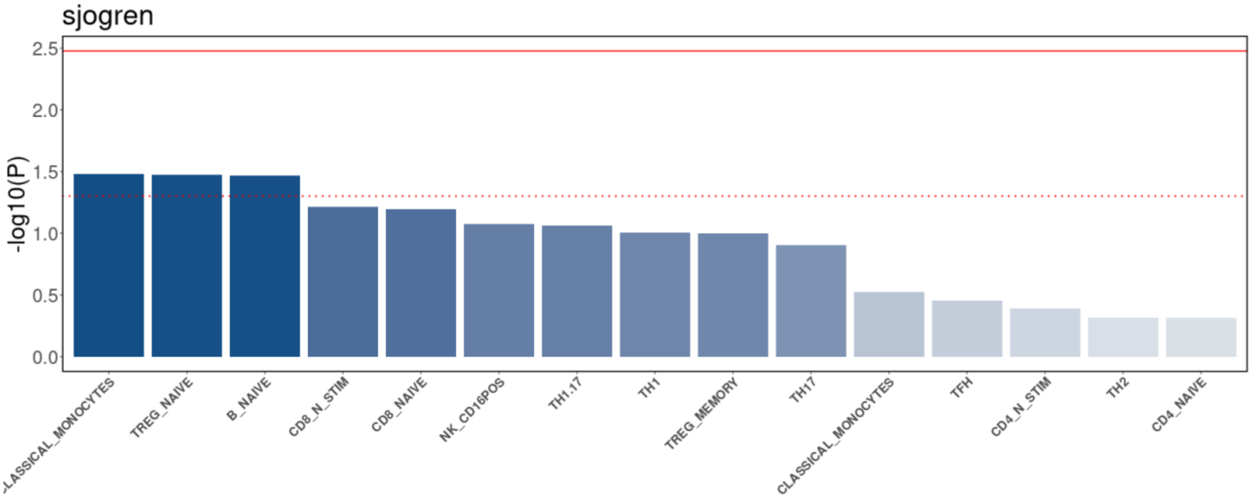

K. Systemic lupus erythematosus.

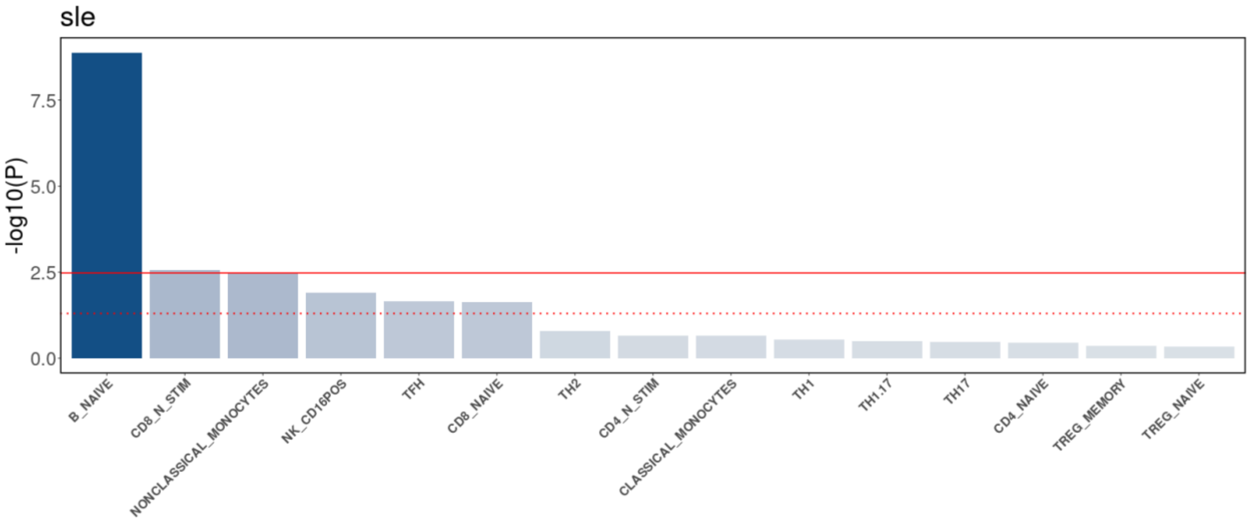

L. Type 1 diabetes.

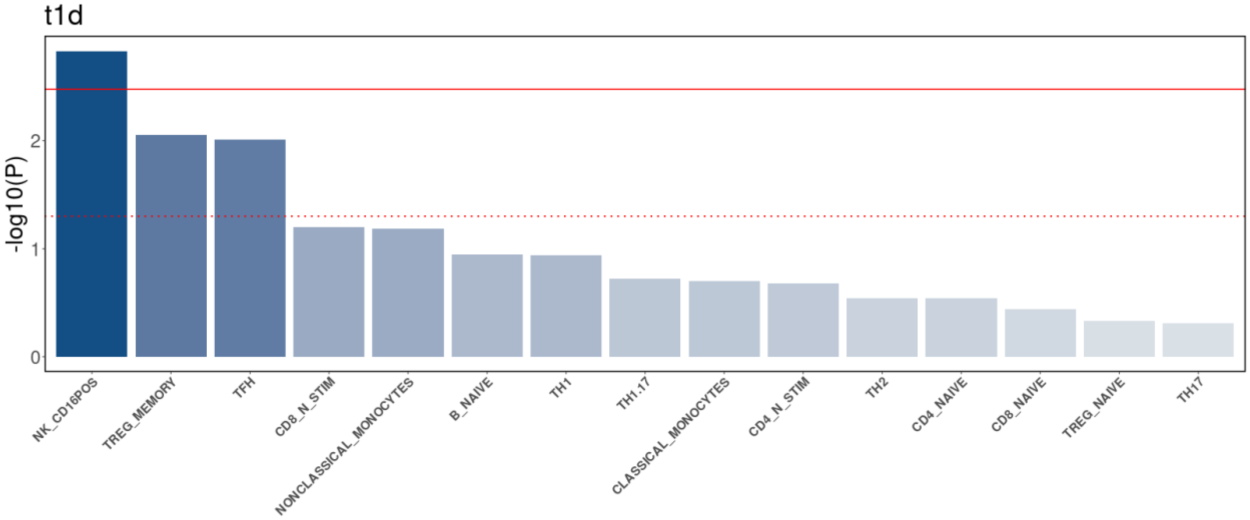

M. Ulcerative colitis.

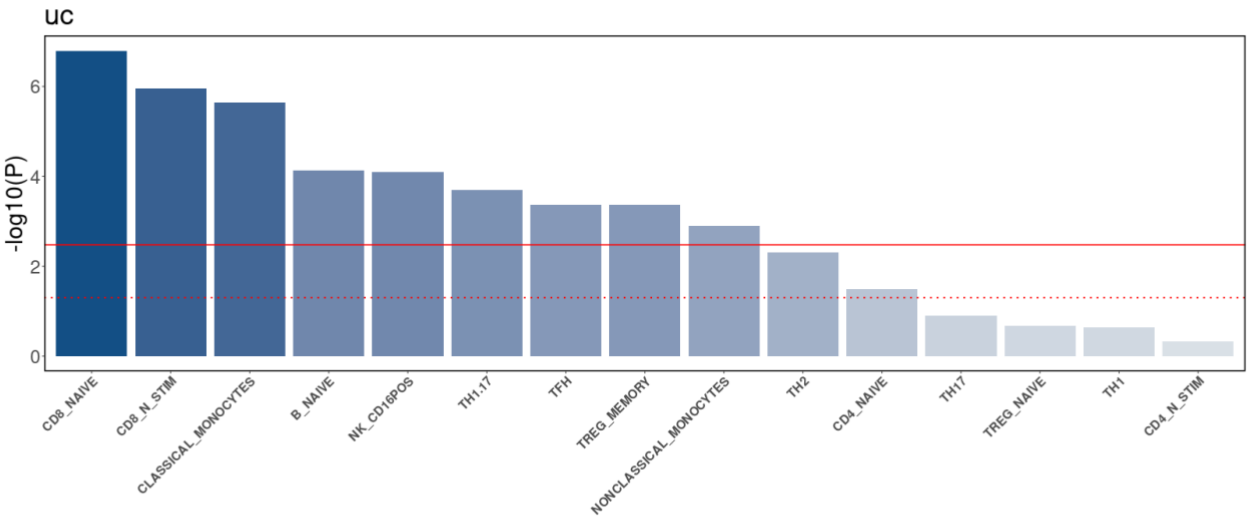

N. Vitiligo

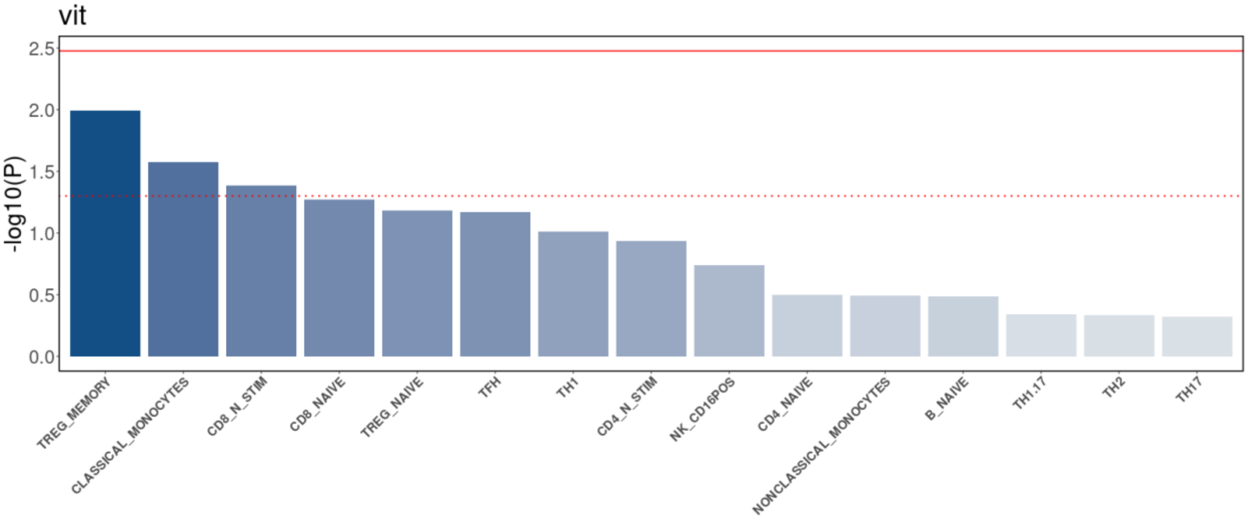

Supplementary Figure 6: Relationship between cell type specific genes, cell type only genotype trait association, and cell type fractions. We seek to understand (A) how the proportion of cell type specific genes (i.e., genes with unusually high expression levels in the cell type) varies with cell type fraction; (B) how the proportion of cell type only GTAs varies with the cell type fraction. We found that rare cell types tend to have more cell type only gene-trait associations and more cell type specific genes.

A.

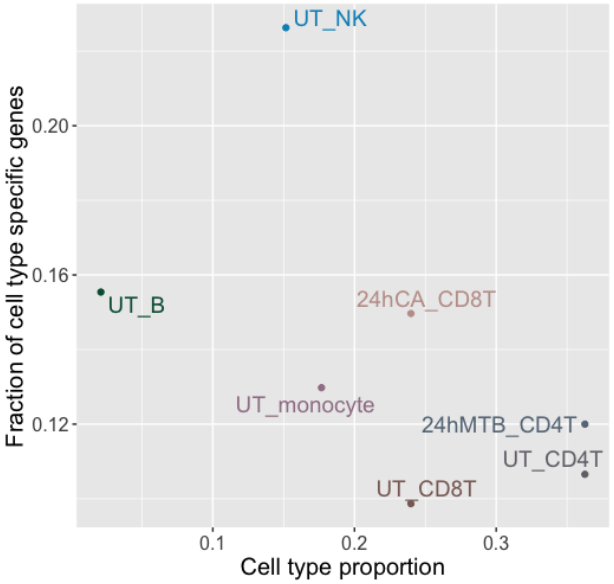

B.

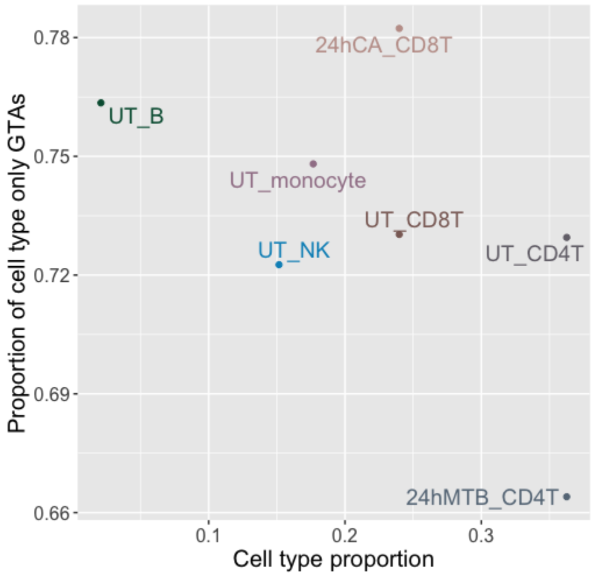

Supplementary Figure 7: Phenotypic effects of predicted gene expression across cell types in 13 autoimmune diseases. Genes in each panel are ordered by their TWAS Z-scores in the whole blood. For each panel, the upper figure shows the TWAS effects across different cell types and whole blood for each gene(Red dashed line represent significant threshold cutoff under two-sided p-value=0.05, red solid line line represent significant threshold cutoff under Bonferroni correction with two-sided p-value=0.05/number of genes), and the lower figure focuses on cell type only gene x trait associations, which zooms-in the middle part of upper figure (i.e., the part between the two red solid lines). Genes in the lower figures have two-sided TWAS p-values > 0.05 in whole blood. The results show that genes with heterogeneous effects across cell types are often missed in whole blood TWAS.

A. **Ankylosing spondylitis**

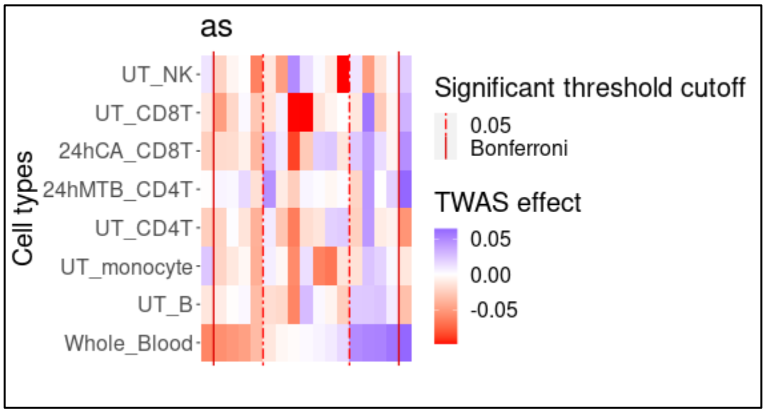

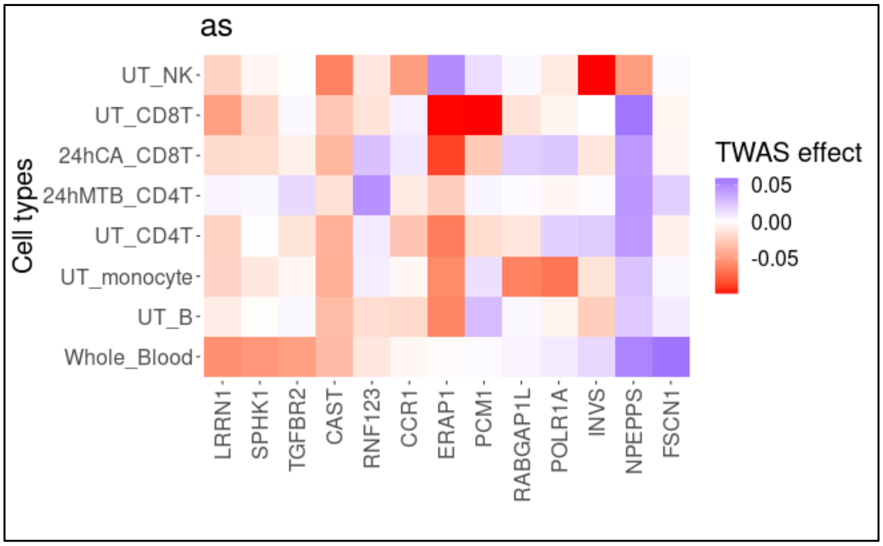

B. Crohn's disease

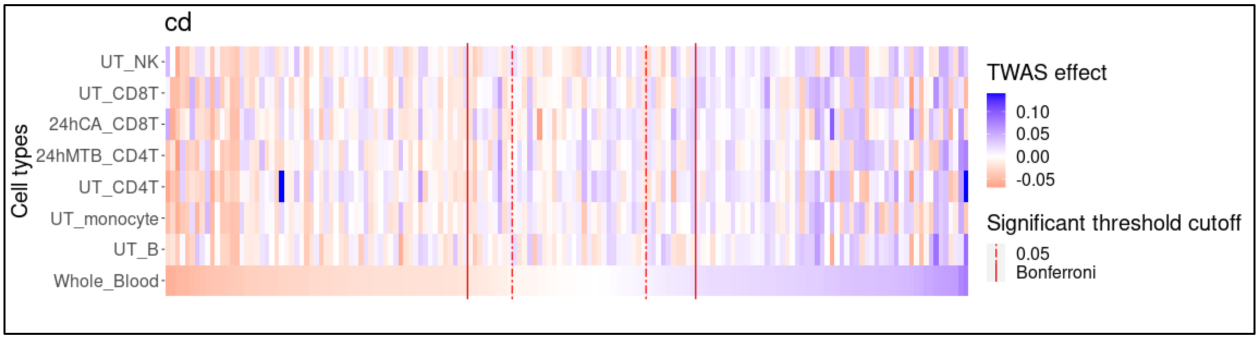

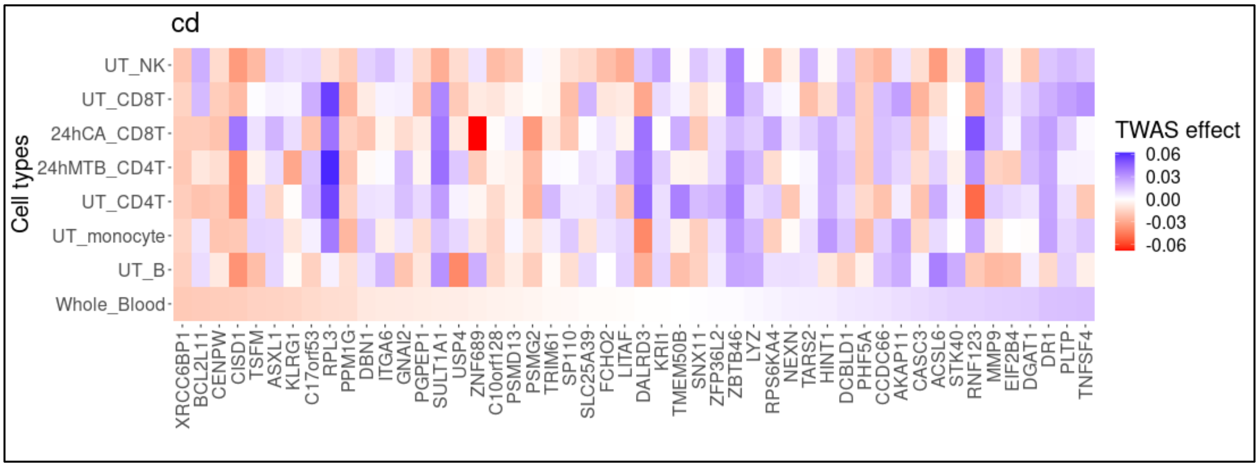

C. Celiac disease

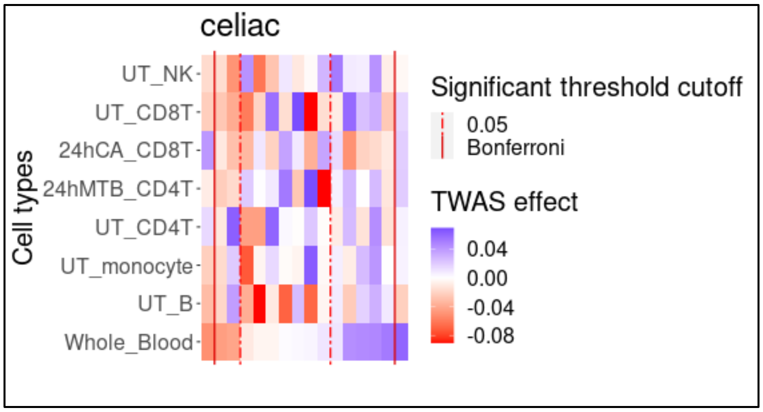

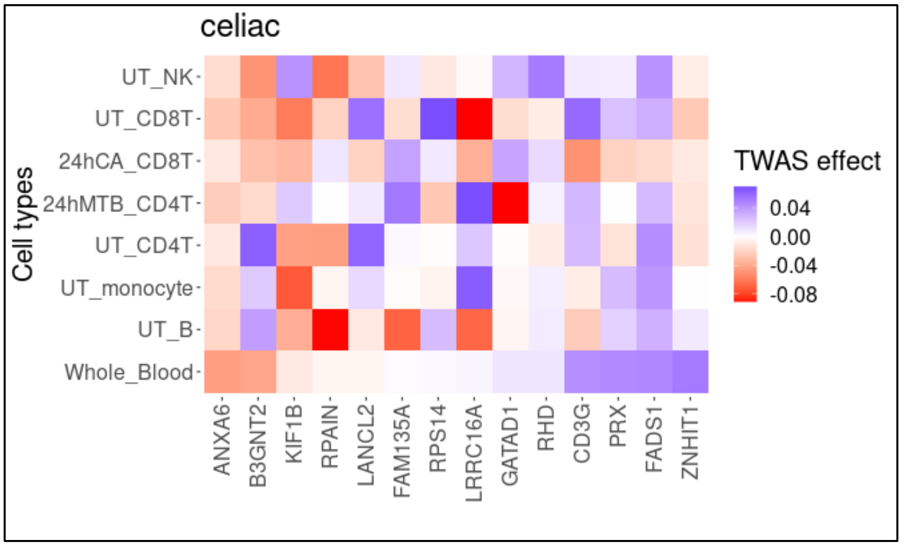

D. ATD-Grave's disease

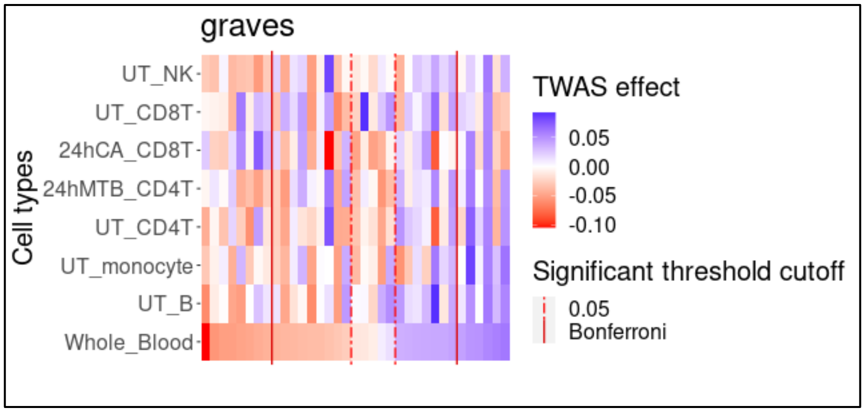

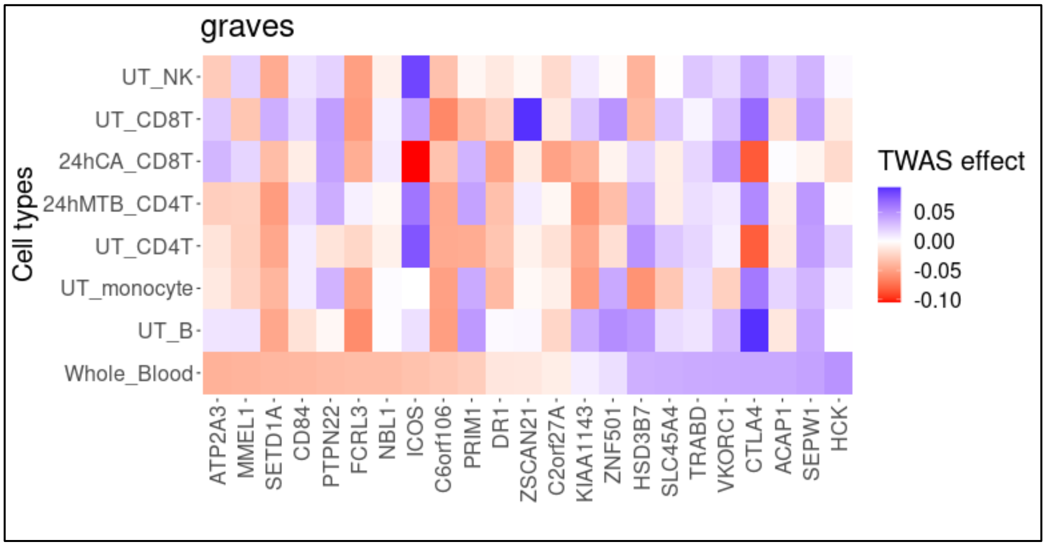

E. ATD-Hashimoto thyroiditis

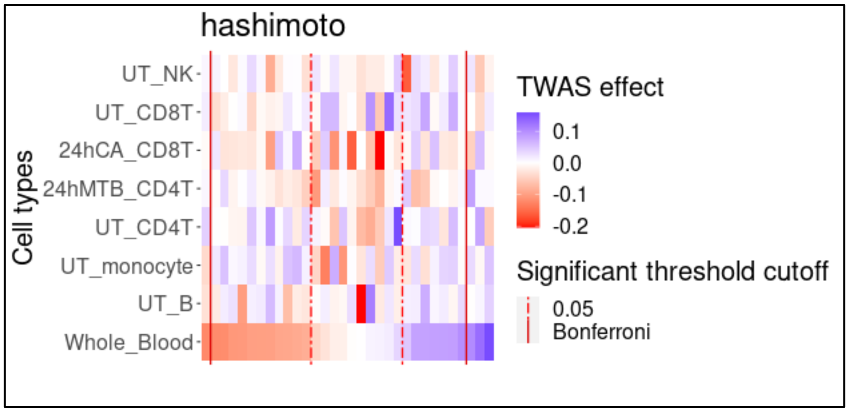

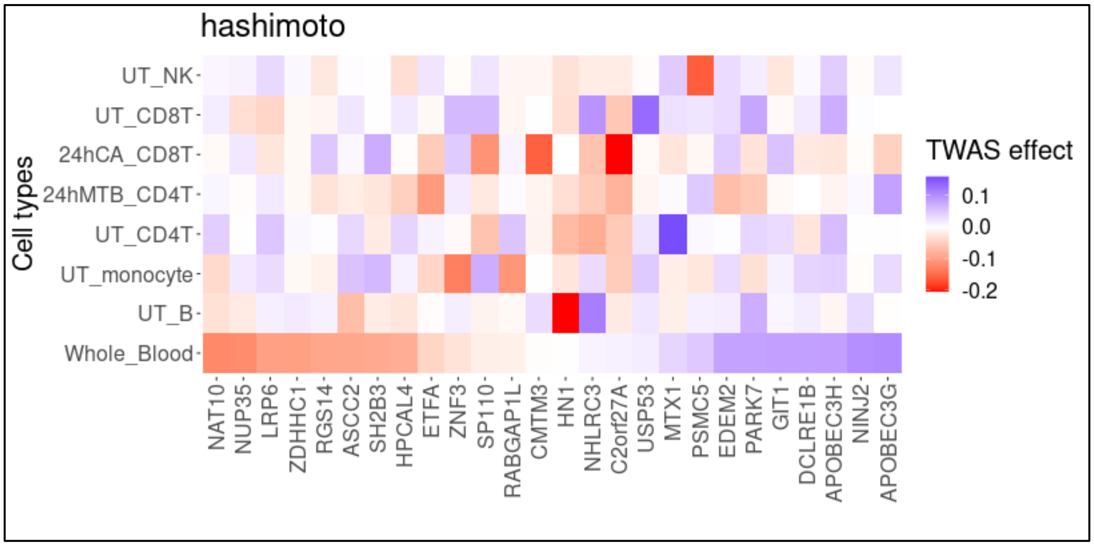

F. Multiple sclerosis

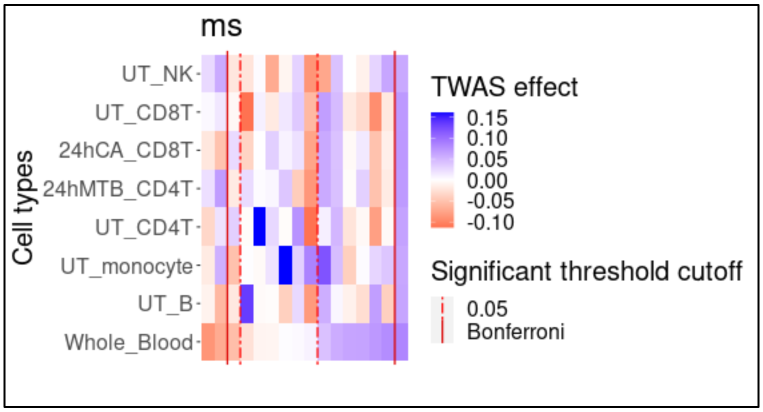

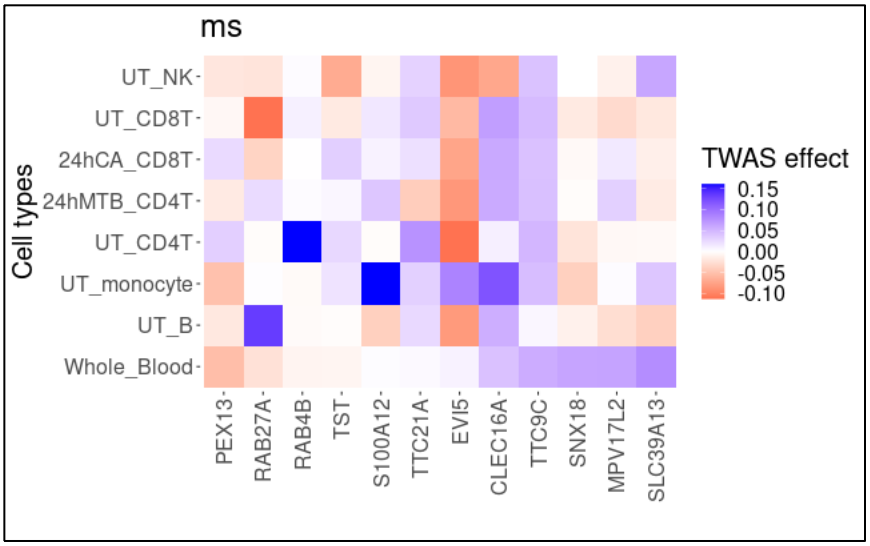

G. Primary biliary cirrhosis

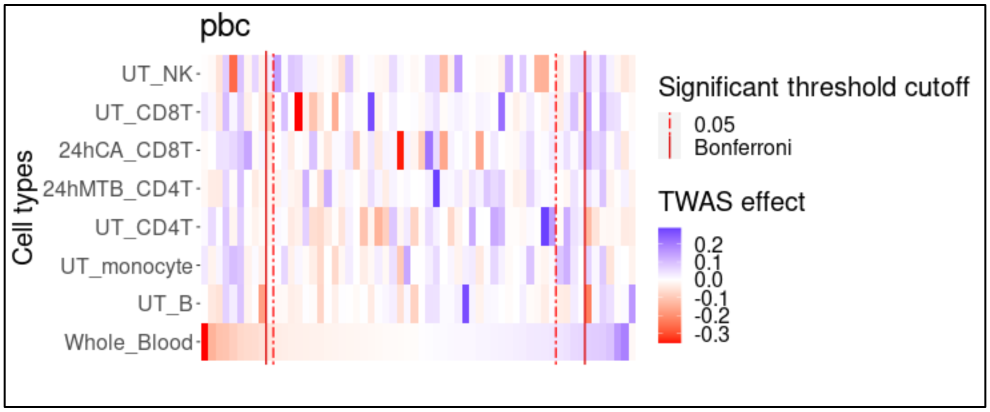

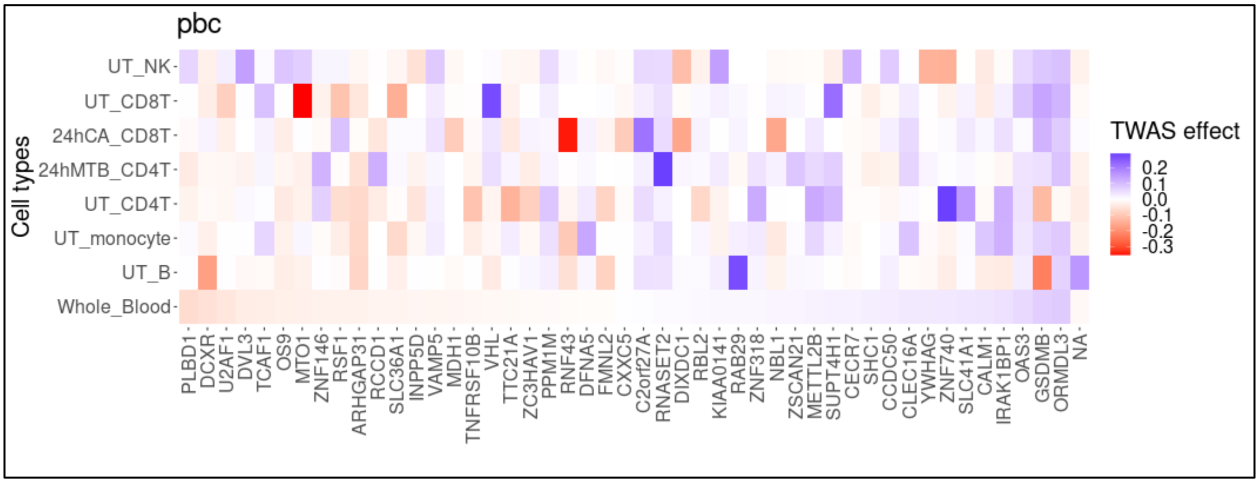

H. Psoriatic arthritis

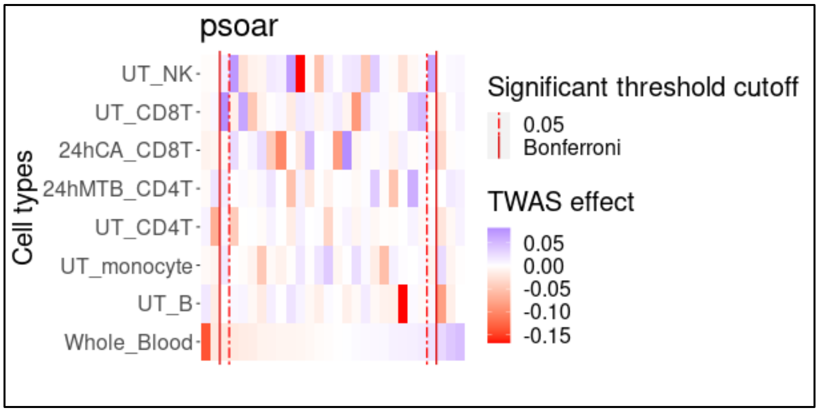

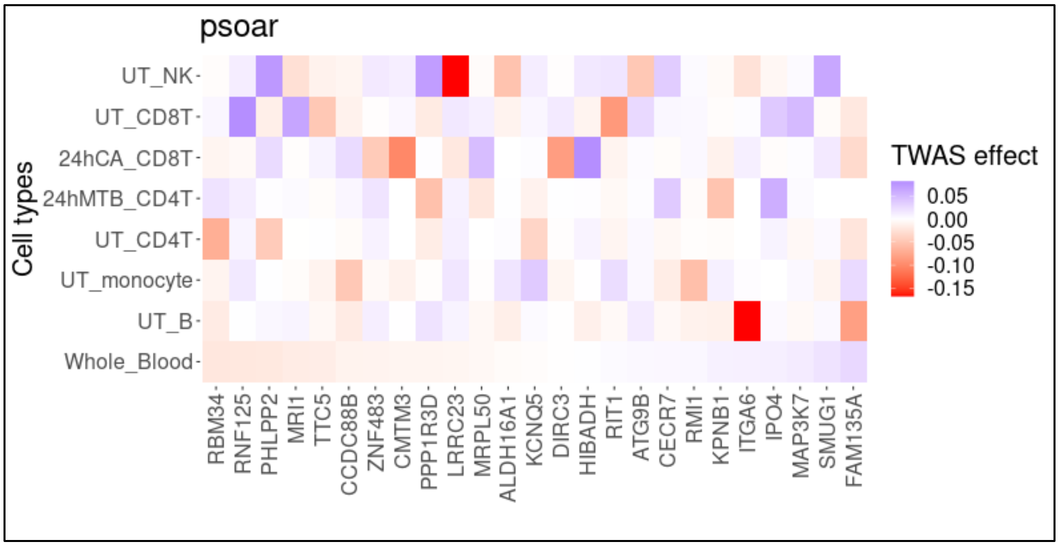

I. Rheumatoid arthritis

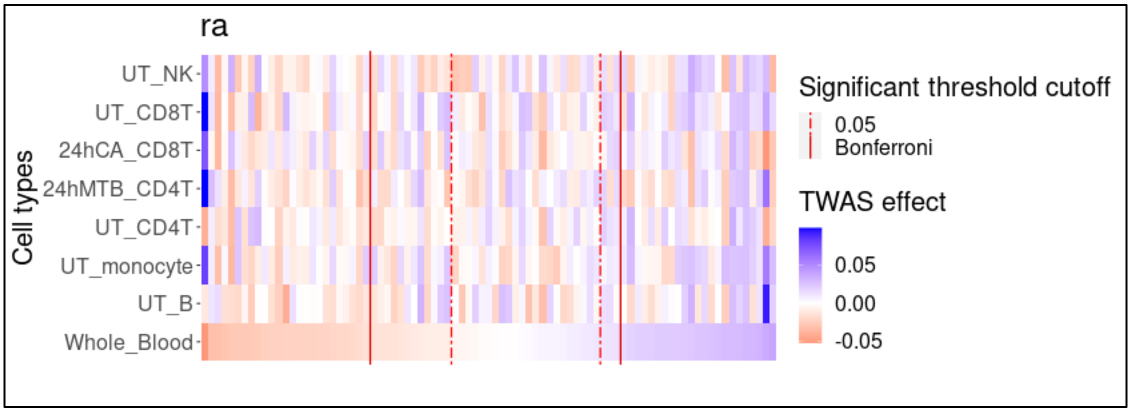

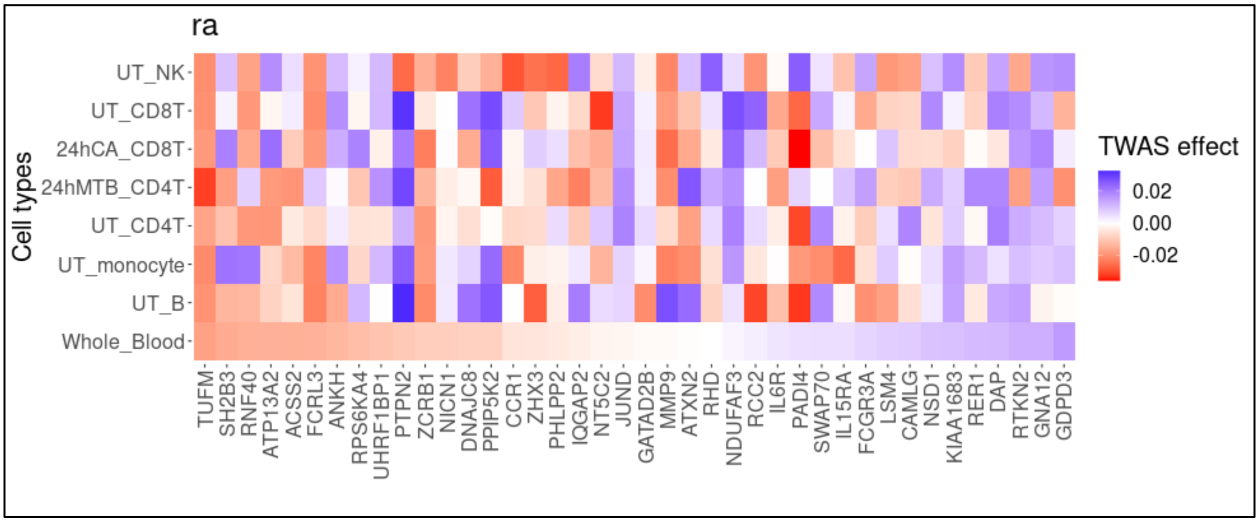

J. Type 1 diabetes

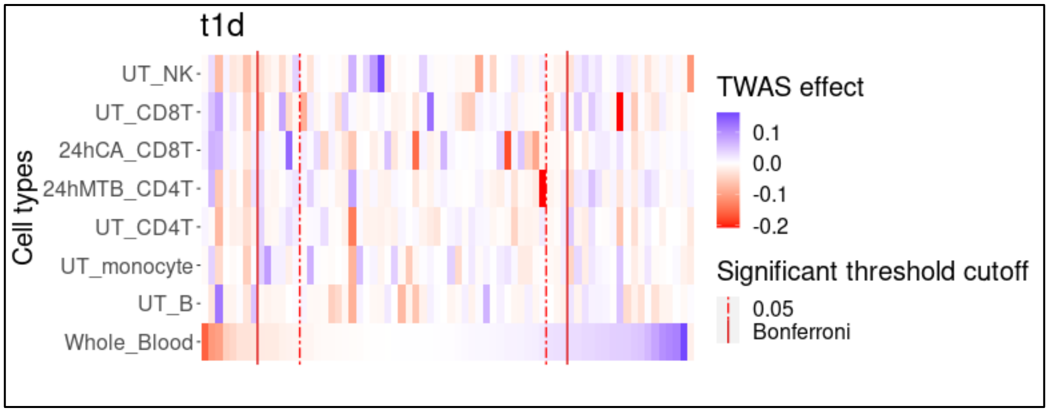

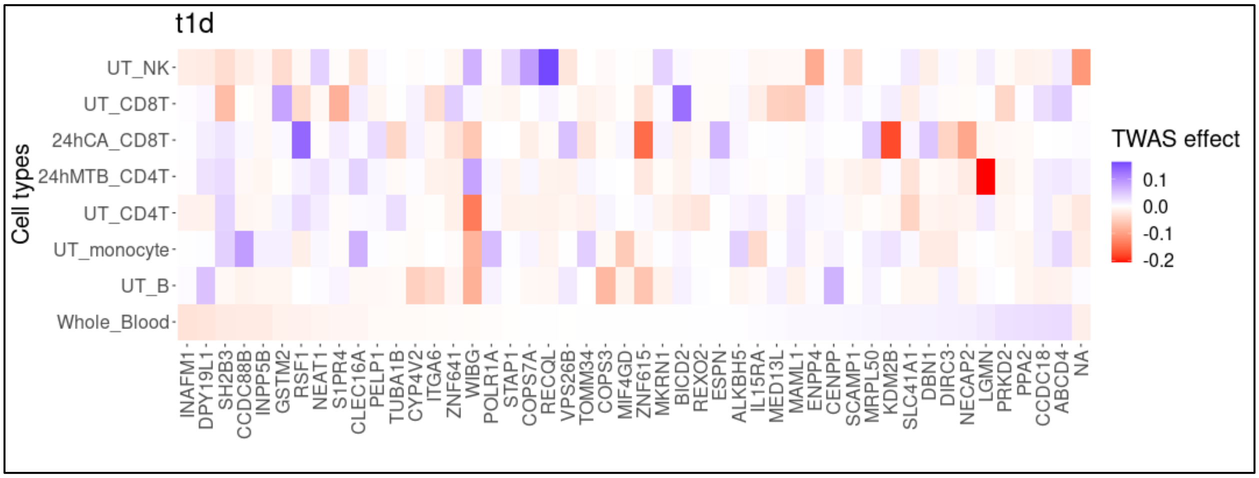

K. Ulcerative colitis

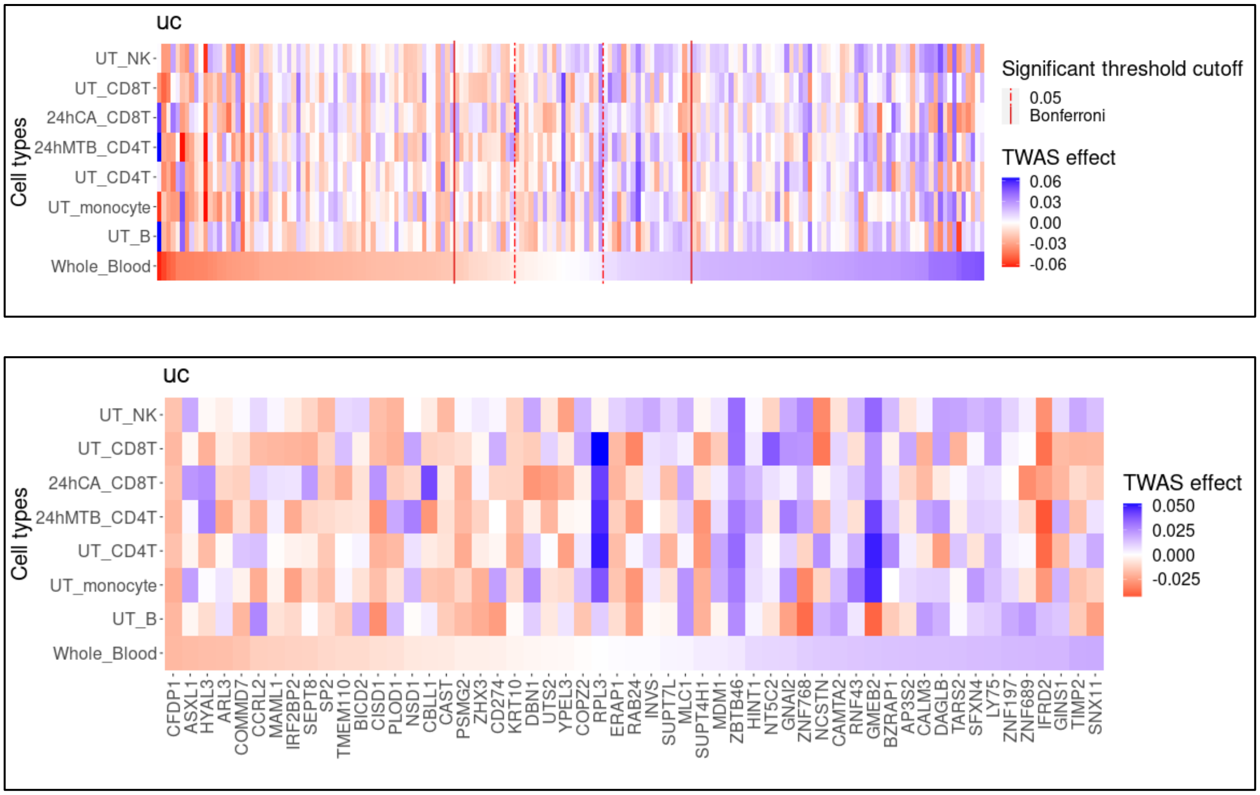

L. Vitiligo

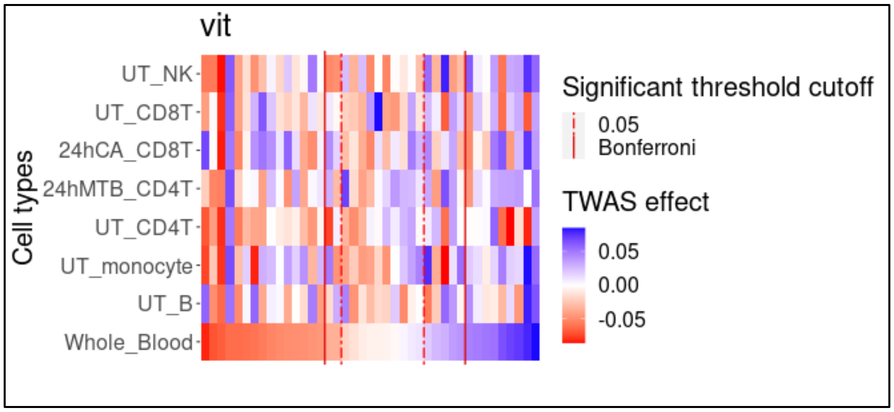
vit
UT_NK
UT_CD8T
24hCA_CD8T
24hMTB_CD4T
UT_CD4T
UT_monocyte
UT_B
Whole_Blood
Cell types
Significant threshold cutoff
0.05
Bonferroni
TWAS effect
0.05
0.00
-0.05

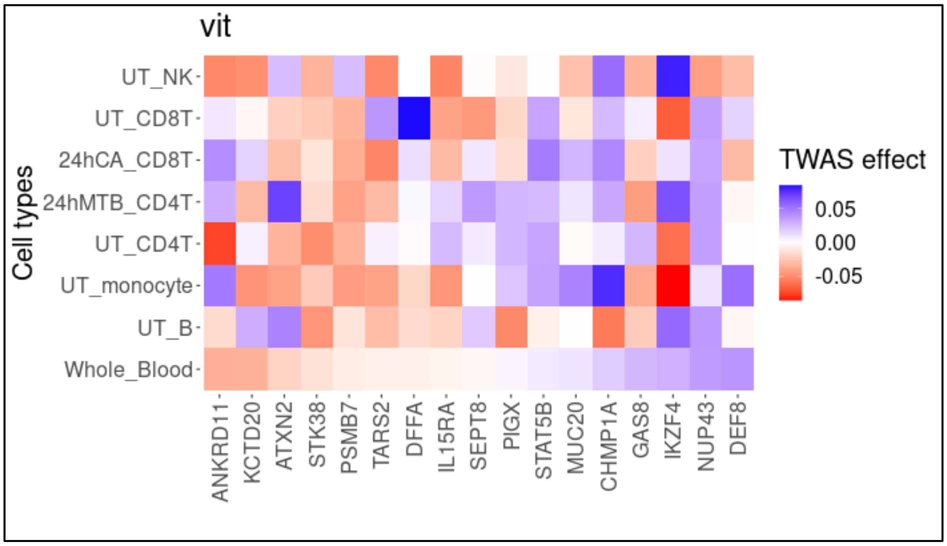
vit
UT_NK
UT_CD8T
24hCA_CD8T
24hMTB_CD4T
UT_CD4T
UT_monocyte
UT_B
Whole_Blood
Cell types
ANKRD11
KCTD20
ATXN2
STK38
PSMB7
TARS2
DFFA
IL15RA
SEPT8
PIGX
STAT5B
MUC20
CHMP1A
GAS8
IKZF4
NUP43
DEF8
TWAS effect
0.05
0.00
-0.05

Supplementary Figure 8: Clustering the cell lines and cell types. We show hierarchical clustering result of different immune cell types and CMAP cell lines. We use it to identify cell lines that mimic the transcriptomic profiles of disease relevant cell types and perform cell type aware drug repurposing.

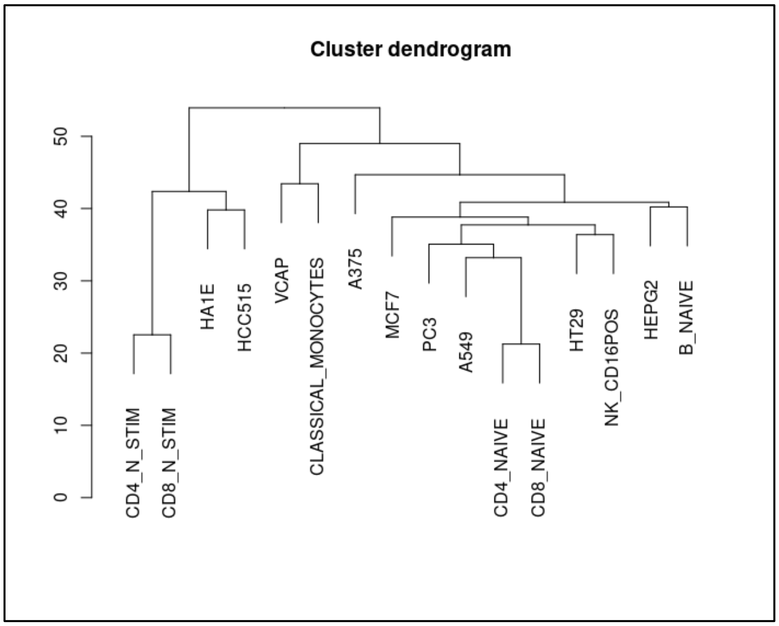

Cell line related tissue: PC3-prostate; VCAP-prostate; A375-skin; A549-lung; HA1E-kidney; HCC515-lung; HT29-large intestine; MCF7-breast; HEPG2-liver

Supplementary Figure 9: Workflow of EXPRESSO.

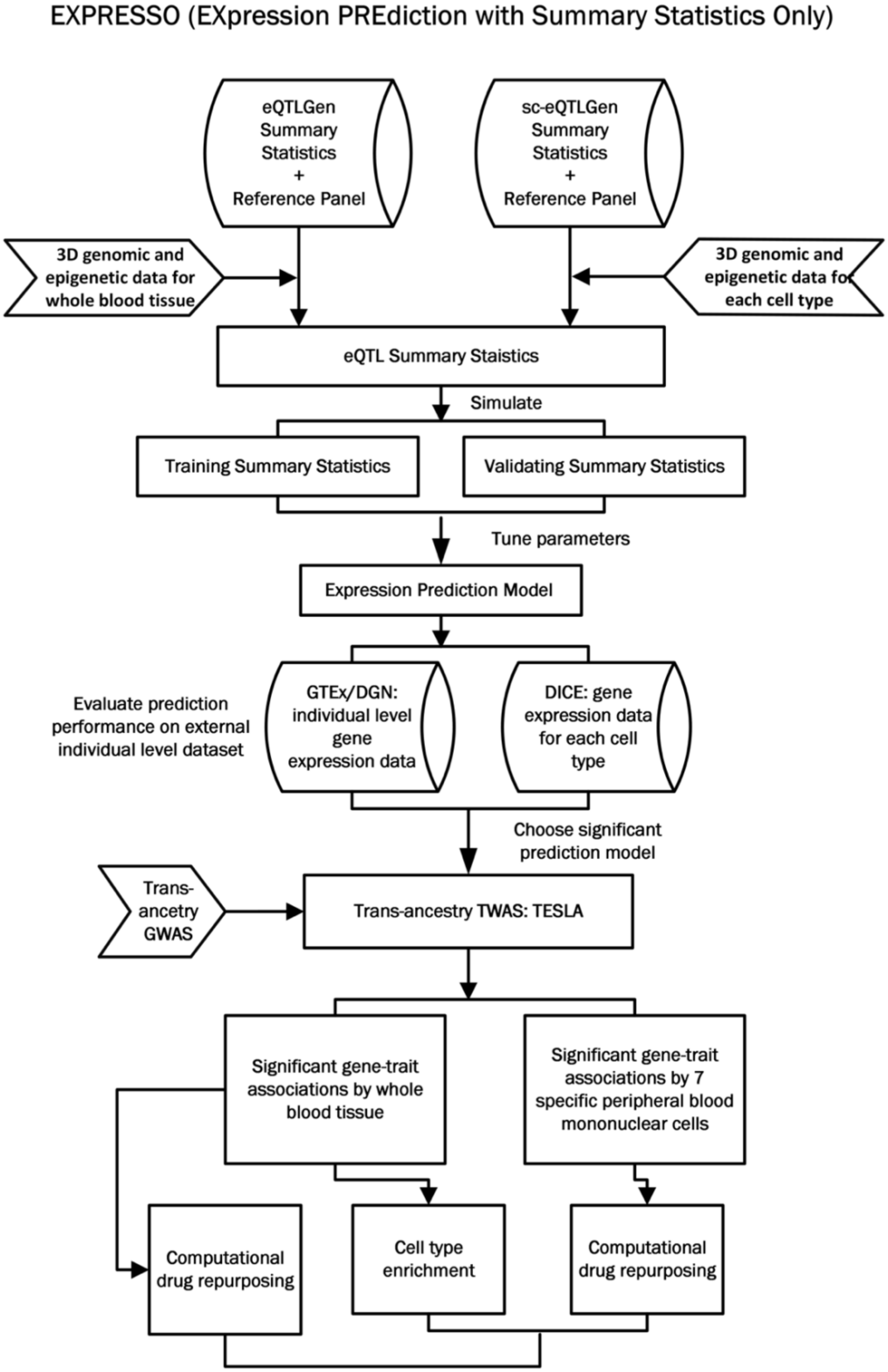

Supplementary Methods

1. Coordinate descent algorithm for fitting EXPRESSO model.

To fit the EXPRESSO model, we develop a coordinate descent algorithm to minimize the loss function and estimate parameters $\boldsymbol{\beta}^{\mathbf{T}} = [\boldsymbol{\beta}_{\mathbf{e}}^{\mathbf{T}}, \boldsymbol{\beta}_{\mathbf{ne}}^{\mathbf{T}}] = \left(\beta_1, \dots, \beta_{p_e}, \beta_{p_e+1}, \dots, \beta_{p_e+p_{ne}}\right)$, where p_e and p_{ne} are the numbers of essential and non-essential predictors. We can estimate $\mathbf{R} = \mathbf{X}^{\mathbf{T}}\mathbf{X}$ from a reference panel of matched ancestry as the eQTL dataset. We can approximate $\mathbf{r} = \mathbf{X}^{\mathbf{T}}\mathbf{y} = \left(r_1, \dots, r_{p_e+n_{pe}}\right)$ using eQTL effect sizes from publicly available datasets such as eQTLGen.

The parameters can be estimated using an iterative coordinate descent algorithm. At step t, we can estimate and update the parameter values as follows:

For the effects of essential predictors, we can estimate the effect estimates $\beta_j^{(t)}, j = 1, \dots, p_e$ by:

$$\beta_j^{(t)} = \begin{cases} \dfrac{sign\left(u_j^{(t)}\right)\left|u_j^{(t)} - \dfrac{n\lambda\phi}{2}\right|}{R_{jj} + \dfrac{n\lambda}{2}} & \text{if } u_j^{(t)} - \dfrac{n\lambda\phi}{2} > 0 \\ 0 & \text{otherwise} \end{cases}$$

For the effects of non-essential predictors, we can estimate the effect estimates $\beta_j^{(t)}, j = p_e + 1, \dots, p_e + p_{ne}$ by:

$$\beta_j^{(t)} = \begin{cases} \dfrac{sign\left(u_j^{(t)}\right)\left|u_j^{(t)} - \dfrac{n\lambda}{2}\right|}{R_{jj} + \dfrac{n\lambda}{2}} & \text{if } u_j^{(t)} - \dfrac{n\lambda}{2} > 0 \\ 0 & \text{otherwise} \end{cases}$$

For $j = 1, \dots, p_e$, $u_j^{(t)}$ corresponds to the "residuals" with respect to essential predictors:

$$u_j^{(t)} = r_j - \sum_{j'=1}^{p_e} I(j' \neq j)\beta_{j'}R_{ij'} - \sum_{j'=p_e+1}^{p} \beta_{j'}R_{ij'}$$

For $j = p_e + 1, \dots, p_e + p_{ne}$, $u_j^{(t)}$ corresponds to the "residuals" with respect to non-essential predictors:

$$u_j^{(t)} = r_j - \sum_{j'=1}^{p_e} \beta_{j'}R_{ij'} - \sum_{j'=p_e+1}^{p} I(j' \neq j)\beta_{j'}R_{ij'}$$

We repeat the procedure until the parameter changes between iterations become less than 1×10^{-8}.

2. Simulation study design.

We perform simulations to compare the accuracy of predicting gene expression and the power for EXPRESSO and other TWAS methods. Briefly, we simulate gene expression values using a modified simulation framework from Nagpal et al[1] , based on real genotypes from three datasets: 1) GTEx (as training data, sample size = 100, 200, 300), 2) UK biobank (for generating large-scale summary statistics, sample size = 20000), 3) Common Mind Consortium (CMC) (as external validation data, sample size = 380). We then simulated GWAS Z-scores following the framework of Feng et al[2] and its adaptations in Khunsriraksakul et al[3].

More specifically, we simulate single-tissue gene expression for gene g based on the linear model:

$$\mathbf{y_g} = \mathbf{X_g}[\boldsymbol{\beta}_{\mathbf{epi}}, \boldsymbol{\beta}_{\mathbf{notepi}}] + \boldsymbol{\varepsilon}_{\mathbf{e}}$$

where $\mathbf{X}_{\boldsymbol{g}}$ is the matrix of normalized genotypes of eQTL variants for gene g, with first M_{epi} columns being essential variants overlapping epigenetic annotation and the remaining M_{notepi} columns being non-essential variants that do not overlap epigenetic annotations. The corresponding genetic effects are given by $\boldsymbol{\beta}_{\mathbf{epi}}$ and $\boldsymbol{\beta}_{\mathbf{notepi}}$. We assume that the gene expression variance explained by cis-regulatory variants is $h_e^2 = \sum_j h_{epi(j)}^2 +$

h^2_{notepi}, where $h^2_{epi(j)}$ is the variance explained by variants in epigenetic annotation j and h^2_{notepi} is the variance explained by non-essential variants. The heritability for each functional category is determined by the overall heritability and the enrichment factor, defined as

$$EF = (h^2_{epi}/M_{epi})/(h^2_{notepi}/M_{notepi})$$

For variants in functional category j, we simulate their effect sizes according to

$$\boldsymbol{\beta}_{\mathbf{epi(j)}} \sim MVN\left(0, \frac{h^2_{epi(j)}}{M_{epi(j)}}\mathbf{I}\right)$$

where $M_{epi(j)}$ is the number of variants that belong to annotation track j, and $\mathbf{I}$ is an identity matrix. In our simulation, we consider four different functional categories for essential variants, i.e., with $epi(1), \dots, epi(4)$ representing H3K27ac mark, H3K4me3 mark, DNase hypersensitive mark, and CTCF mark. The effects for non-essential variants are simulated according to normal distributions $\boldsymbol{\beta}_{\boldsymbol{notepi}} \sim MVN(0, h^2_{notepi}/M_{notepi}\boldsymbol{I}_{\boldsymbol{M_{notepi}}})$.

The residual error is assumed to follow $\boldsymbol{\varepsilon_e} \sim MVN(0, (1 - h^2_e)\mathbf{I}_{\boldsymbol{N_{train}}})$ where N_{train} is the training sample sizes. Finally, we simulate GWAS Z-scores according to the multivariate normal distribution $\mathbf{Z} \sim MVN(\boldsymbol{\Sigma_G} \times \sqrt{N_{GWAS} \times h^2_p} \times [\boldsymbol{\beta}_{\mathbf{epi}}, \boldsymbol{\beta}_{\mathbf{notepi}}], \boldsymbol{\Sigma}_{\mathbf{G}})$ where $\boldsymbol{\Sigma}_{\mathbf{G}}$ is the LD matrix of the cis-SNPs (calculated from 5,000 randomly selected samples of European ancestry from UK Biobank), N_{GWAS} is the GWAS sample size (set to 500,000), and h^2_p is the phenotypic variance explained by the gene expression.

To simulate gene expression levels from single tissues, we consider the following scenarios by varying
1) the number of causal SNPs ($n_{causal} \in \{2, 8, 32, 64\}$).
2) the proportion of causal SNPs in the epigenomic regions ($p_{causal-epi} \in \{0.4, 0.8, 1\}$).
3) the window containing causal SNPs ($w \in$ ±1Mb, ±250kb, 3D window, mixed), where mixed refers to the simulation that randomly selects one window from ±1Mb, ±250kb, and 3D windows each gene.
4) the expression and phenotypic variance $(h^2_{e_1}, h^2_{e_2}, h^2_{e_3}, h^2_{e_4}, h^2_p) \in$
$\{(0.0103, 0.0073, 0.0060, 0.0012, 0.05), (0.0208, 0.0146, 0.0121, 0.0025, 0.01), (0.0416,$
$0.0292, 0.0242, 0.0050, 0.005)\}$. The values are based on estimates reported in previous studies[4].
5) epigenomic enrichment factor ($EF \in \{2, 4\}$).
6) the training sample sizes (100, 200, and 300). For summary statistics-based methods, we also consider n = 20000, as eQTL summary statistics are often available from much larger datasets.

Besides, we also consider additional simulation to evaluate the scenarios where annotation does not affect variant causality and effect sizes (Supplementary Data 3), to assess the impact of annotations on power. We summarize the power by averaging over 1000 replicates in each scenario.

To simulate the gene expression for multi-tissue method, we start with a causal tissue where the genetic variants directly influence expression levels. These causal genetic variants may also influence the gene expression levels of other non-causal tissues with weaker effects, because of genetic correlations between gene expression levels of causal and other tissues. We use all 513 individuals in GTEx and retain the same sample sizes of different tissues. We choose brain frontal cortex (BA9) tissue as a causal tissue (n_{BA9} = 118) and simulate the gene expression level as in single tissue analysis, i.e.,

$$\mathbf{y}_{\mathbf{g_{causal}}} = \mathbf{X_g}[\boldsymbol{\beta}_{\mathbf{causal\_epi}}, \boldsymbol{\beta}_{\mathbf{causal\_notepi}}] + \boldsymbol{\varepsilon}_{\mathbf{e}}$$

We assume that the genetic effects in other tissues (which we call "correlated tissues") may be genetically correlated with the causal issue. We vary the number of correlated tissues (N_{corr}) and the genetic correlation (ρ) of eQTL effects between causal tissue and correlated tissues in our simulation. We generate the eQTL effects in correlated tissues by

$$\boldsymbol{\beta}_{\mathbf{corr}} = \boldsymbol{MVN}(\rho \left[\boldsymbol{\beta}_{\mathbf{causal_{epi}}}, \boldsymbol{\beta}_{\mathbf{causal_{notepi}}} \right], (1-\rho^2) \times h_e^2 \times \mathbf{I}_{\mathbf{N_{corr}}})$$

Gene expression levels of correlated tissues are simulated according to

$$\mathbf{y}_{\mathbf{g_{corr}}} = \mathbf{X_g}\boldsymbol{\beta}_{\mathbf{corr}} + \boldsymbol{\varepsilon}_{\mathbf{e}_*}$$

Where $\boldsymbol{\varepsilon}_{\mathbf{e}_*} \sim MVN\left(\mathbf{0}, \boldsymbol{diag}\left(\sqrt{1-h_e^2}\right) \times \boldsymbol{\Sigma} \times \boldsymbol{diag}\left(\sqrt{1-h_e^2}\right)\right)$, and $\boldsymbol{\Sigma}$ is the residual correlation matrix among gene expression levels across tissues. For uncorrelated tissue, we simulate the gene expression in the same way but set $\rho = 0$. We consider the following five scenarios with (ρ, N_{corr}) values being (0.3, 0), (0.3, 24), (0.7 24), (0.3, 47), and (0.7, 47). For each replicate, we randomly choose other parameters as in single tissue simulation. We report the power by averaging over 1000 replicates for each pair of parameter values.

Given the simulated expression levels, we simulate GWAS Z-scores following the framework of Khunsriraksakul et al[3] by randomly sampling from multivariate normal distribution as below:

$$\mathbf{Z} \sim MVN\left(\boldsymbol{\Sigma}_{\mathbf{G}} \times \sqrt{N_{GWAS} \times h_p^2} \times \left[\boldsymbol{\beta}_{\mathbf{causal_{epi}}}, \boldsymbol{\beta}_{\mathbf{causal_{notepi}}}\right], \boldsymbol{\Sigma}_{\mathbf{G}}\right)$$

3. Simulation results.

We perform extensive simulations to evaluate EXPRESSO-PVS, EXPRESSO-MSE and other methods, including TWAS methods that use individual level genotype and expression information, i.e., PUMICE[3], PrediXcan[5], FUSION[6], TIGAR[1], EpiXcan[7], and multi-tissue method UTMOST[8], summary statistics based PRS methods PUMAS[9], LDpred2[10] and two summary statistics based TWAS methods SUMMIT[11] and OTTERS[12], which incorporates four summary statistics based PRS methods pruning and thresholding (P+T)[13], SDPR[14], PRScs[15] and LASSOSUM[16]. We considered scenarios with different sample sizes, proportions of causal variants, window size parameters (with either regulatory regions defined by linear windows relative to gene start and end sites or defined by 3D genomes), and proportions of gene expression heritability explained by essential variants in different categories[4] and the enrichment factor of heritability in essential variants. We focus on three metrics in the comparison:

1) the number of significant models where the predicted and measured gene expressions are significantly correlated with Pearson's correlation coefficient (R) > 0.1 and the p-value of R is less than 0.05.
2) Pearson correlation (R) between predicted and measured gene expression levels, and
3) the power for TWAS, which is defined as the fraction of genes with significant gene expression prediction model and significant TWAS p-values (i.e., $p < 5 \times 10^{-5}$, the Bonferroni threshold for testing 1000 simulated genes).

Based on the training dataset, we compare the prediction accuracy in independently simulated test datasets for the following scenarios:

(1) EXPRESSO-PVS, EXPRESSO-MSE, SUMMIT, OTTERS [which combines pruning and thresholding with p-value cutoffs of 0.001 (P+0.001) or 0.05 (P+0.05), SDPR, PRScs, LASSOSUM], PUMAS and LDpred2 trained on small-scale summary statistics (n=100, 200, and 300);
(2) the same set of methods trained on large-scale summary statistics (n=20000).
(3) Five single tissue TWAS methods (PUMICE, PrediXcan, FUSION, TIGAR and EpiXcan) trained on small-scale individual level data (n=100, 200, 300);
(4) UTMOST (a multi-tissue TWAS method) trained on simulated individual-level data from 48 tissues. When simulating expression levels from multiple tissues, we keep the same sample size of each tissue as in GTEx. We consider scenarios with different proportions of shared causal eQTL variants between tissue types. We consider a single causal tissue (BA9, n_{BA9}= 118) where eQTLs influence gene expression levels. We also consider different

numbers of "correlated tissues", where the eQTL effects are genetically correlated with that of causal tissue and influence the expression level with weaker effects.

We first compared EXPRESSO-PVS with PRS methods applied to the same set of large-scale eQTL summary statistics. We observe that EXPRESSO attains the highest prediction accuracy, proportion of significant models, and power. Compared to EXPRESSO-MSE, SUMMIT, P+0.001, P+0.05, SDPR, PRScs, LASSOSUM, PUMAS and LDpred2, we observed 8.95%, 21.96%, 26.74%, 26.81%, 11.82%, 11.58%, 19.28%, 25.72% and 45.92% gain in prediction accuracy, and 6.00%, 20.86%, 18.91%, 18.88%, 11.01%, 11.45%, 15.72%, 22.56% and 30.51% gain in the number of significant models. The improved prediction accuracy also translates to a gain of 7.55%, 10.68%, 16.24%, 16.27%, 11.48%, 8.79%, 11.44%, 31.41% and 37.01% in TWAS power (Supplementary Figure 1). In these scenarios, we note that the improvement of EXPRESSO-PVS over the second-best method (PRScs) (8.79%) is bigger than the improvement of PRScs over the third- (SDPR 2.43%) and fourth-best methods (LASSOSUM 2.47%).

On the other hand, as eQTL summary statistics are often available from much larger sample sizes, we also apply summary statistics-based methods to datasets with larger sample sizes (n=20,000) and compare them with methods requiring individual level information (with n = 100, 200, or 300). Not surprisingly, EXPRESSO-PVS trained on large summary statistics datasets outperforms methods relying on smaller individual level data even more. When compared with PUMICE, PrediXcan, FUSION, TIGAR, EpiXcan trained on the dataset with n = 300, EXPRESSO-PVS leads to an average increase of 59.31%, 84.60%, 92.35%, 93.16% and 61.26% for the number of significant prediction models and similar increases in the median of Pearson's correlation and the power of TWAS (Supplementary Figure 1).

In most applications, EXPRESSO-PVS and other summary statistics-based methods will be applied to eQTL summary statistics from much larger sample sizes. Yet, to further investigate methodological properties of EXPRESSO-PVS, we compare EXPRESSO-PVS with TWAS methods that rely on individual level data, when they analyze the same set of samples. EXPRESSO-PVS only needs eQTL summary statistics from the same set of individuals (n = 100, 200, or 300) as input. Yet, it still yields similar performance to PUMICE and EpiXcan and outperforms other TWAS methods (i.e., PrediXcan, FUSION, and TIGAR), which do not incorporate 3D genome information or epigenetic data. EXPRESSO-PVS and PUMICE minimize the same loss function. Yet, PUMICE relies on individual level data and uses cross validation to select tuning parameters. Comparing EXPRESSO-PVS with PUMICE allows us to assess the impact of tuning parameter selection methods. EXPRESSO-PVS outperforms PUMICE in scenarios with smaller sample sizes (n=100, 200), larger numbers of causal variants (32 or 64), or lower gene expression heritability (h_e^2=0.025, 0.05). In these three scenarios, the average prediction accuracy increases of EXPRESSO-PVS relative to PUMICE are 4.27%, 9.84%, 9.51% respectively. EXPRESSO-PVS performs slightly worse than PUMICE when the training sample size is large (e.g., n = 300), when there is a smaller number of causal variants (e.g., the number of causal variants is 2 or 8), or when the gene expression heritability is high (e.g., $h_e^2 = 0.1$). The average prediction accuracy decreases compared to PUMICE, however, are more modest, i.e., -2.41%, -7.83%, -3.91% (Supplementary Data 4-5). Compared to multi-tissue TWAS method (UTMOST), we find that EXPRESSO-PVS outperforms UTMOST when the training sample size is high (i.e., 200 or 300) across all five scenarios in simulation. When the training sample size is small (e.g., n = 100), EXPRESSO still outperforms UTMOST when the genetic correlation between causal and correlated tissues is low (i.e., 0 or 0.3) (Supplementary Figure 1 and Supplementary Data 6), as it is more difficult for UTMOST to borrow information across tissues in these scenarios.

We also consider scenarios where variant causality and effect sizes do not depend on annotations. Given that EXPRESSO can integrate annotation information, these scenarios where annotation information do not affect variant causality are worst-case scenarios for EXPRESSO. Importantly, in those scenarios, EXPRESSO-PVS still

performs better or at least comparably to alternative methods, which demonstrate the robustness of methods and the consistent improvement of power. Specifically, when the number of causal variants equals to 2, the TWAS power of EXPRESSO-PVS is 68.7% which is substantially higher than the second-best method PRScs (55.7%). We also added the scenario when the number of causal variant equals to 4. The power of EXPRESSO-PVS is 65.0% which is still higher than PRScs (59.2%). With annotation information, the power improvement of EXPRESSO-PVS over PRScs can be much higher (69.0% vs 53.2%).

It is clear from this comparison that EXPRESSO-PVS already outperforms methods based on individual level data, even when they all analyze the same set of individuals. As eQTL summary statistics are often available in much larger sample sizes, EXPRESSO-PVS can have an even bigger advantage and stands out as the clear choice.

Supplementary References.

1. Nagpal, S. *et al.* TIGAR: An Improved Bayesian Tool for Transcriptomic Data Imputation Enhances Gene Mapping of Complex Traits. *Am J Hum Genet* **105**, 258-266 (2019).
2. Feng, H. *et al.* Leveraging expression from multiple tissues using sparse canonical correlation analysis and aggregate tests improves the power of transcriptome-wide association studies. *PLoS Genet* **17**, e1008973 (2021).
3. Khunsriraksakul, C. *et al.* Integrating 3D genomic and epigenomic data to enhance target gene discovery and drug repurposing in transcriptome-wide association studies. *Nat Commun* **13**, 3258 (2022).
4. Finucane, H.K. *et al.* Partitioning heritability by functional annotation using genome-wide association summary statistics. *Nat Genet* **47**, 1228-35 (2015).
5. Gamazon, E.R. *et al.* A gene-based association method for mapping traits using reference transcriptome data. *Nat Genet* **47**, 1091-8 (2015).
6. Gusev, A. *et al.* Integrative approaches for large-scale transcriptome-wide association studies. *Nat Genet* **48**, 245-52 (2016).
7. Zhang, W. *et al.* Integrative transcriptome imputation reveals tissue-specific and shared biological mechanisms mediating susceptibility to complex traits. *Nat Commun* **10**, 3834 (2019).
8. Hu, Y. *et al.* A statistical framework for cross-tissue transcriptome-wide association analysis. *Nat Genet* **51**, 568-576 (2019).
9. Zhao, Z. *et al.* PUMAS: fine-tuning polygenic risk scores with GWAS summary statistics. *Genome Biol* **22**, 257 (2021).
10. Prive, F., Arbel, J. & Vilhjalmsson, B.J. LDpred2: better, faster, stronger. *Bioinformatics* (2020).
11. Zhang, Z., Bae, Y.E., Bradley, J.R., Wu, L. & Wu, C. SUMMIT: An integrative approach for better transcriptomic data imputation improves causal gene identification. *Nat Commun* **13**, 6336 (2022).
12. Dai, Q. *et al.* OTTERS: a powerful TWAS framework leveraging summary-level reference data. *Nat Commun* **14**, 1271 (2023).
13. International Schizophrenia, C. *et al.* Common polygenic variation contributes to risk of schizophrenia and bipolar disorder. *Nature* **460**, 748-52 (2009).
14. Zhou, G. & Zhao, H. A fast and robust Bayesian nonparametric method for prediction of complex traits using summary statistics. *PLoS Genet* **17**, e1009697 (2021).
15. Ge, T., Chen, C.Y., Ni, Y., Feng, Y.A. & Smoller, J.W. Polygenic prediction via Bayesian regression and continuous shrinkage priors. *Nat Commun* **10**, 1776 (2019).
16. Mak, T.S.H., Porsch, R.M., Choi, S.W., Zhou, X. & Sham, P.C. Polygenic scores via penalized regression on summary statistics. *Genet Epidemiol* **41**, 469-480 (2017).
