# Supplementary material for: Integrating single cell expression quantitative trait loci summary statistics to understand complex trait risk genes
Source: Nat Commun. 2024 May 20;15:4260. doi: 10.1038/s41467-024-48143-1 (PMC11519974; doi:10.1038/s41467-024-48143-1)
Supplement: Supplementary file 3 — Description of Additional Supplementary Files [file 41467_2024_48143_MOESM3_ESM.pdf]

File Name: Supplementary Data 1
Description: Simulation comparison of methods trained using large-scale summary statistics (n=20000). (a) Pearson correlation between measured and predicted gene expression levels. Median Pearson correlation (r) is reported.; (b) Proportion of significant models (i.e., Pearson correlation > 0.1 with two-sided p-value < 0.05). (c) TWAS power and standard deviation measured by the proportion of genes with two-sided TWAS p-values < 0.05/the number of genes tested. Results are averaged over scenarios with different numbers of causal variants, heritability, and enrichment factor of essential variants.

File Name: Supplementary Data 2
Description: By scenario simulation comparison of summary statistics methods trained on large-scale summary statistics (n=20000). (a) Pearson correlation between measured and predicted gene expression levels. Median Pearson correlation (r) is reported.; (b) Proportion of significant models (Pearson correlation > 0.1 with two-sided p-value < 0.05). (c) TWAS power measured by the proportion of genes with two-sided TWAS p-values < 0.05/the number of genes tested. Results are shown for all scenarios with different parameter settings.

File Name: Supplementary Data 3
Description: Simulation comparison of methods trained on large-scale summary statistics (n=20000) when variant effect and annotation do not depend on annotations. (a) Pearson correlation between measured and predicted gene expression levels. Median Pearson correlation (r) is reported.; (b) Proportion of significant models (i.e., Pearson correlation > 0.1 with two-sided p-value < 0.05). (c) TWAS power and standard deviation measured by the proportion of genes with two-sided TWAS p-values < 0.05/the number of genes tested. Results are shown for all scenarios with different parameter settings.

File Name: Supplementary Data 4
Description: The summary of simulation comparison of summary statistics methods and individual data level methods trained on small-scale summary statistics (n=100, 200, 300). (a) Pearson correlation between measured and predicted gene expression levels. Median Pearson correlation (r) is reported.; (b) Proportion of significant models (i.e., Pearson correlation > 0.1 with two-sided p-value < 0.05). (c) TWAS power and and standard deviation measured by the proportion of genes with two-sided TWAS p-values < 0.05/the number of genes tested. Results are averaged over scenarios with different numbers of causal variants, heritability, and enrichment factor of essential variants.

File Name: Supplementary Data 5
Description: Simulation comparison of summary statistics methods and individual data level methods trained on large-scale summary statistics (n=100, 200, 300). (a) Pearson correlation between measured and predicted gene expression levels. Median Pearson correlation (r) is reported.; (b) Proportion of significant models (Pearson correlation > 0.1 with two-sided p-value < 0.05). (c) TWAS power and and standard deviation measured by the proportion of genes whose two-sided TWAS p-values < 0.05/the number of genes tested. Results are shown for all scenario with different parameter settings.

File Name: Supplementary Data 6

Description: Simulation comparison of summary statistics methods and individual data level methods trained on large-scale summary statistics (n=100, 200, 300). (a) Pearson correlation between measured and predicted gene expression levels. Median Pearson correlation (r) is reported.; (b) Proportion of significant models (Pearson correlation > 0.1 with two-sided p-value < 0.05). (c) TWAS power and standard deviation measured by the proportion of genes whose two-sided TWAS p-values < 0.05/the number of genes tested. Results are shown for all scenario with different parameter settings.

File Name: Supplementary Data 7
Description: Comparison of the accuracy of gene expression prediction models using external tests datasets. We train the prediction model using eQTLGen/GTEx/DGN, GTEx individual level data, or GTEx summary statistics from the same set of individuals. We validate the predicition accuracy in DGN. We report the number of significant models (i.e., models with the Pearson correlation between measured and predicted gene expression > .1 and two-sided p < 0.05) and the median of Pearson correlations.

File Name: Supplementary Data 8
Description: Comparison of prediction accuracy for EXPRESSO and other methods in 7 cell types using DICE dataset as validation. We compare (1) the proportion of significant models (i.e., the Pearson's correlation (r) between measured and predicted gene expression > .1 with two-sided p < 0.05); (2) the median r among the union of significant models.

File Name: Supplementary Data 9
Description: GWAS datasets for 14 autoimmune traits. We list the disease names, their publication sources, ancestry of the datasets, and sample sizes.

File Name: Supplementary Data 10
Description: The numbers of loci, novel loci, known loci, median of chi-square statistics in known loci between EXPRESSO and other TWAS methods based on whole blood results. We define loci iteratively: We first rank significant genes(two-sided p-value <0.05/number of significant models) by their p-values, from small to large. To define the first locus, we consider 1 million basepair window surrounding the start and end position of the top gene and remove any overlapping genes from the list. We then repeat the process to define the locus surrounding the most significant genes remaining in the list. We repeat the process until we exhaust all genes in the list. We define a locus as novel if it is 1 million base pairs away from reported GWAS hits, and a locus as known if it is within 1 million base pairs of any known GWAS hits of the trait.

File Name: Supplementary Data 11
Description: The numbers of identified loci, novel loci, known loci for each disease identified using EXPRESSO and other TWAS methods in whole blood.

File Name: Supplementary Data 12
Description: Genomic control factor for TWAS statistics across all methods in eQTLGen/GTEx/DGN, DGN, GTEx and sc-eQTLGen analysis.

File Name: Supplementary Data 13
Description: Whole blood and brain cell type enrichment p-values using TWAS results based on EXPRESSO across 14 autoimmune diseases. We highlight the significant whole blood cell types (two-sided $p<0.0033$, the Bonferroni threshold for testing 15 immune cell types) and brain cell types (two-sided $p<0.05/17=0.0029$) with yellow color. As all diseases (with the exception of multiple sclerosis) are not related to brain cell types, brain cell types are used as negative controls. Reassuringly, virtually no brain cell types show enrichment with TWAS hits of autoimmune diseases.

File Name: Supplementary Data 14
Description: The number of loci and significant GTAs identified by EXPRESSO and other methods across different cell types.

File Name: Supplementary Data 15
Description: Proportion of cell type specific essential variants used in the prediction model for cell type only gene-trait associations and gene-trait associations also identified in whole blood. We define the cell type specific essential variants as the ones that overlap cell type specific epigenetic marks. The gene expression prediction model for cell type only gene x trait associations (GTAs) tend to contain a bigger fraction of cell type specific essential variants.

File Name: Supplementary Data 16
Description: Fine-mapping results of TWAS loci in whole blood tissue and 7 cell types across 14 autoimmune diseases.

File Name: Supplementary Data 17
Description: Pathway enrichment results using Enrichr across all traits and cell types.

File Name: Supplementary Data 18
Description: We summarized the effects of genetically predicted gene expression across different cell types and whole blood tissue for genes with significant p-values in at least one cell type (two-sided p-value < 0.05/number of significant models). This table includes the effects of genetically predicted gene expression levels plotted in Figure 4 and Supplementary Figure 4.

File Name: Supplementary Data 19
Description: Cell type enrichment p-value using EXPRESSO and other twas methods. We also report the number of significantly enriched cell types with two-sided p-value < 0.05 and significantly enriched cell types with two-sided p-value < 0.0033 (Bonferroni correction for testing 15 cell types). EXPRESSO generally yields stronger enrichment results compared to alternative methods.

File Name: Supplementary Data 20
Description: Drug repruposing analysis and drug enrichment analysis using CARDE and whole blood TWAS results. (a) We report average τ scores for each drug and for each disease using results from cell type aware drug repurposing analysis (CADRE) and results from drug repurposing analysis with whole blood TWAS. (b) For each known drug, we conduct enrichment analysis assessing whether the

drug pathways are enriched with TWAS hits from whole blood and with TWAS hits from disease relevant cell types as identified by CADRE. P-values are shown for the enrichment analysis. The results show that the approved drugs are more enriched with TWAS hits from disease relevant cell types than from whole blood, which demonstrates the advantage of our cell type aware drug repurposing pipelines.
