# Supplementary material for: Integrating single cell expression quantitative trait loci summary statistics to understand complex trait risk genes
Source: Nat Commun. 2024 May 20;15:4260. doi: 10.1038/s41467-024-48143-1 (PMC11519974; doi:10.1038/s41467-024-48143-1)
Supplement: Supplementary file 5 — Reporting Summary [file 41467_2024_48143_MOESM5_ESM.pdf]

nature portfolio

Corresponding author(s): Laura Carrel, Dajiang J. Liu, Bibo Jiang.

Last updated by author(s): Apr 1, 2024

Reporting Summary

Nature Portfolio wishes to improve the reproducibility of the work that we publish. This form provides structure for consistency and transparency in reporting. For further information on Nature Portfolio policies, see our Editorial Policies and the Editorial Policy Checklist.

Statistics

For all statistical analyses, confirm that the following items are present in the figure legend, table legend, main text, or Methods section.

n/a	Confirmed	
☐	☒	The exact sample size (*n*) for each experimental group/condition, given as a discrete number and unit of measurement
☒	☐	A statement on whether measurements were taken from distinct samples or whether the same sample was measured repeatedly
☐	☒	The statistical test(s) used AND whether they are one- or two-sided<br>*Only common tests should be described solely by name; describe more complex techniques in the Methods section.*
☐	☒	A description of all covariates tested
☐	☒	A description of any assumptions or corrections, such as tests of normality and adjustment for multiple comparisons
☐	☒	A full description of the statistical parameters including central tendency (e.g. means) or other basic estimates (e.g. regression coefficient) AND variation (e.g. standard deviation) or associated estimates of uncertainty (e.g. confidence intervals)
☐	☒	For null hypothesis testing, the test statistic (e.g. *F*, *t*, *r*) with confidence intervals, effect sizes, degrees of freedom and *P* value noted<br>*Give P values as exact values whenever suitable.*
☒	☐	For Bayesian analysis, information on the choice of priors and Markov chain Monte Carlo settings
☒	☐	For hierarchical and complex designs, identification of the appropriate level for tests and full reporting of outcomes
☐	☒	Estimates of effect sizes (e.g. Cohen's *d*, Pearson's *r*), indicating how they were calculated

Our web collection on statistics for biologists contains articles on many of the points above.

Software and code

Policy information about availability of computer code

Data collection	We did not collect new data and no software was used for data collection.
Data analysis	EXPRESSO software: https://github.com/LidaWangPSU/EXPRESSO;<br>PUMICE software: https://github.com/ckhunsr1/PUMICE;<br>PrediXcan software: https://github.com/hakyimlab/MetaXcan;<br>FUSION software: https://github.com/gusevlab/fusion_twas;<br>EpiXcan software: https://bitbucket.org/roussoslab/epixcan/src/master/;<br>TIGAR software: https://github.com/yanglab-emory/TIGAR;<br>CTIMP (UTMOST) software: https://github.com/yiminghu/CTIMP;<br>TESLA software: https://github.com/funfunchen/rareGWAMA;<br>LDpred software: https://privefl.github.io/bigsnpr/articles/LDpred2.html;<br>LASSOSUM software: https://github.com/tshmak/lassosum;<br>PUMAS software: https://github.com/qlu-lab/PUMAS;<br>PLINK software: https://www.cog-genomics.org/plink;<br>Bedtools software: https://bedtools.readthedocs.io/en/latest;<br>CMap software: https://clue.io/;<br>OTTERS software: https://github.com/daiqile96/OTTERS;<br>SUMMIT software: https://github.com/ChongWuLab/SUMMIT.

For manuscripts utilizing custom algorithms or software that are central to the research but not yet described in published literature, software must be made available to editors and reviewers. We strongly encourage code deposition in a community repository (e.g. GitHub). See the Nature Portfolio guidelines for submitting code & software for further information.

Data

Policy information about availability of data

All manuscripts must include a data availability statement. This statement should provide the following information, where applicable:

- Accession codes, unique identifiers, or web links for publicly available datasets
- A description of any restrictions on data availability
- For clinical datasets or third party data, please ensure that the statement adheres to our policy

The eQTLGen summary statistics are publicly available from https://eqtlgen.org/cis-eqtls.html. The sc-eQTLGen summary statistics are available from https://eqtlgen.org/sc/datasets/1m-scbloodnl-eqtls.html. GTEx V7 data can be obtained from dbGaP study accession phs000424.v7.p2. DGN data can be requested at https://www.nimhgenetics.org/request-access/how-to-request-access under "Depression Genes and Networks study (D. Levinson, PI)". DICE dataset can be requested through dbGaP accession number phs001703.v1.p1. Epigenomic data were obtained from http://screen.encodeproject.org. 3D genomic data were obtained from http://3dgenome.org. Cell type aware computational drug repurposing analysis was conducted on CLUE Drug Repurposing Hub, which can be accessed at https://clue.io/repurposing-app. GWAS summary statistics files are publicly available and Pubmed ID for each study is provided in supplementary table 9.

Research involving human participants, their data, or biological material

Policy information about studies with human participants or human data. See also policy information about sex, gender (identity/presentation), and sexual orientation and race, ethnicity and racism.

Reporting on sex and gender	NA
Reporting on race, ethnicity, or other socially relevant groupings	NA
Population characteristics	NA
Recruitment	NA
Ethics oversight	NA

Note that full information on the approval of the study protocol must also be provided in the manuscript.

Field-specific reporting

Please select the one below that is the best fit for your research. If you are not sure, read the appropriate sections before making your selection.

☒ Life sciences ☐ Behavioural & social sciences ☐ Ecological, evolutionary & environmental sciences

For a reference copy of the document with all sections, see nature.com/documents/nr-reporting-summary-flat.pdf

Life sciences study design

All studies must disclose on these points even when the disclosure is negative.

Sample size	We used eQTLGen(N=31684) and sc-eQTLGen (N=120) summary statistics to generate our gene expression prediction models. No sample size calculation was performed.
Data exclusions	No data were excluded from the analyses.
Replication	Whole blood tissue gene expression prediction models were validated in GTEx V7 European sample (N=303) and Depression Genes and Networks study(DGN) European sample (N=873). Cell type spesific gene expression prediction models were validated in Database of Immune Cell Expression (DICE, n=91).
Randomization	Randomization is not applicable. Because we focus on existing eQTL summary statistics and don't need to collect and process individual level data.
Blinding	Blinding is not applicable. Because we focus on existing eQTL summary statistics and don't need to collect and process individual level data.

Reporting for specific materials, systems and methods

We require information from authors about some types of materials, experimental systems and methods used in many studies. Here, indicate whether each material, system or method listed is relevant to your study. If you are not sure if a list item applies to your research, read the appropriate section before selecting a response.

Materials & experimental systems

n/a	Involved in the study	
☒	☐	Antibodies
☒	☐	Eukaryotic cell lines
☒	☐	Palaeontology and archaeology
☒	☐	Animals and other organisms
☒	☐	Clinical data
☒	☐	Dual use research of concern
☒	☐	Plants

Methods

n/a	Involved in the study	
☒	☐	ChIP-seq
☒	☐	Flow cytometry
☒	☐	MRI-based neuroimaging
